# Promoting ethical and reproducible cell culture: implementing animal-free alternatives to teaching in molecular and cell biology

**DOI:** 10.3389/ftox.2025.1670513

**Published:** 2025-10-01

**Authors:** Alexandra Nessar, Viola Röhrs, Mathias Ziersch, Ahmed S. M. Ali, Julia Moradi, Anke Kurreck, Johanna Berg, Jens Kurreck

**Affiliations:** ^1^ Chair of Applied Biochemistry, Institute of Biotechnology, Technische Universität Berlin, Berlin, Germany; ^2^ Chair of Bioprocess Engineering, Institute of Biotechnology, Technische Universität Berlin, Berlin, Germany; ^3^ BioNukleo GmbH, Berlin, Germany; ^4^ Research, Transfer and Startup Center, Anhalt University of Applied Science, Köthen, Germany

**Keywords:** chemically defined media, FBS-free, fetal bovine serum, HeLa cells, animal free research

## Abstract

The widespread use of fetal bovine serum (FBS) and other animal-derived reagents in cell culture raises ethical concerns and scientific limitations, including batch variability and undefined composition. To address these challenges and promote the adoption of xeno-free, human-relevant methods, we developed a graduate-level laboratory course based on animal-free workflows. The curriculum covers key molecular and cell biology techniques: HeLa cell culture and passaging, transfection, RNA interference (RNAi), quantitative RT-PCR (qRT-PCR), dual-luciferase assays, and Western blotting, using reagents selected to exclude animal-derived components. A chemically defined medium (CDM) was optimized for robust HeLa cell growth in the absence of FBS, and recombinant TrypLE was implemented as a substitute for porcine trypsin. Validated non-animal-derived antibodies are also introduced. The course has been successfully piloted and provides a scalable, ethical framework for modern bioscience education. A detailed, open-access protocol enables replication and dissemination. This initiative equips students with practical skills and educational foundation in animal-free methodologies, supporting a shift toward reproducible and ethically responsible biomedical research.

## 1 Introduction

**FIGURE 1:**
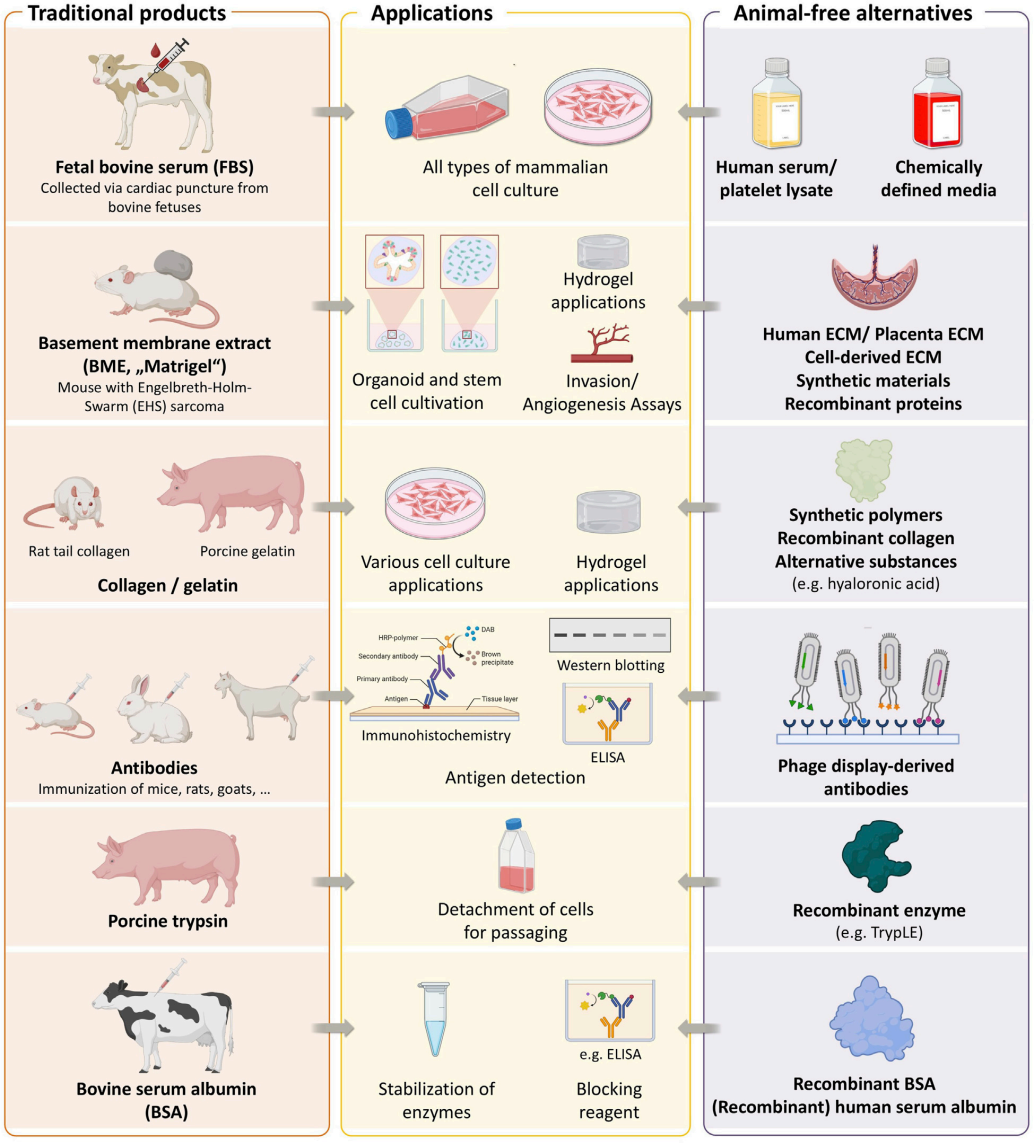
Key animal-derived components used in biomedical research. The figure summarizes some commonly used materials, their biological sources, and available non-animal alternatives. Created with BioRender.com.

The establishment of the first immortalized human cell line, HeLa, in 1951 marked a major milestone in biomedical research. Another significant development was the introduction of fetal bovine serum (FBS) as a cell culture supplement in 1958 (Puck et al., 1958), which greatly facilitated the cultivation of mammalian cells. Since then, countless cell lines have been developed, enabling substantial advances in *vitro* studies of human physiology and disease, and finding widespread application in basic research, toxicity testing, and the production of biologics. It has become standard practice to supplement culture media with 5%–20% FBS. Beyond FBS for supporting cell proliferation (van der Valk et al., 2018), numerous other animal-derived reagents are routinely employed in biomedical research, including porcine trypsin for cell detaching and passaging (Helgason and Miller, 2013), basement membrane extracts (BMEs, also known under the brand name Matrigel) for stem cell and 3D culture applications (Benton et al., 2014), bovine serum albumin (BSA) for protein stabilization, animal-derived antibodies for immunodetection (Gray et al., 2020), and collagen or gelatin for hydrogel formation in tissue engineering (Berg and Kurreck, 2021; Duarte et al., 2023) (Figure 1). Despite their widespread use, the continued reliance on animal products is increasingly questioned, driven by both ethical concerns over their origin and scientific limitations related to their undefined and variable composition (van der Valk et al., 2010; Gstraunthaler, 2003).

FBS exemplifies these issues. Harvested from bovine fetuses, usually via cardiac puncture during the slaughter of cows found to be pregnant at slaughter, a process that raises significant animal welfare concerns. Estimates suggest that millions of fetuses are used annually to meet global demand (Subbiahanadar Chelladurai et al., 2021; Weber et al., 2025). From a scientific standpoint, FBS is an undefined and variable mixture whose composition can differ based on batch, season, and geographical source (van der Valk et al., 2018; Liu et al., 2023). This variability contributes to reproducibility issues and undermines efforts to standardize cell-based methods. A feature article in *Nature* identified FBS as a major contributor to variability in cell culture experiments, emphasizing the need for standardized and well-defined alternatives (Baker, 2016).

In addition, the use of FBS undermines efforts to enhance the human relevance of biomedical research. By introducing non-human biological material into human cell systems, FBS creates chimeric models that may distort physiological responses and limit the translational validity of experimental findings. This concern is underscored by the high attrition rate in drug development, where approximately 90% of candidates that perform well in preclinical studies, including *in vitro* and animal models, ultimately fail in human clinical trials (Wong et al., 2019; Marshall et al., 2023), often due to interspecies differences and the inadequacy of existing model systems.

To address these concerns, chemically defined media (CDMs) have been developed as serum-free and, in most cases, animal-free alternatives. These media are precisely formulated and contain components such as insulin, transferrin, selenium, as well as essential nutrients tailored to specific cell types (van der Valk et al., 2010; Wijerathna-Yapa et al., 2025). More recently, a universal medium has been developed that supports the growth of many cell types tested so far including, but not limited to, breast cancer JIMT-1 and MDA-MB-231 cells, colon cancer CaCo-2 cells, pancreatic cancer MiaPaCa-2 cells and the mouse L929 cell line. (Rafnsdottir et al., 2023). The exact composition of the medium was reported, and detailed preparation instructions were provided in a subsequent publication (Weber et al., 2024). This innovation holds the potential to be transformative, and despite its complex formulation and silghtly higher production costs more and more groups started using it. In some cases, it may be more practical to culture cells in a simpler, cell type–specific medium. Such media can be more cost-efficient and easier to prepare and - depending on the research question - may offer advantages by containing only factors relevant to the cell type of interest. For several cell lines, including HeLa, animal-free media are commercially available. However, these media are often expensive, and their exact composition is typically undisclosed for proprietary reasons, which undermines efforts to achieve reproducible cell culture.

Parallel innovations have produced animal-free alternatives for other common reagents applied in cell and molecular biology. This includes using recombinant proteins such as TrypLE, an animal-free alternative to porcine trypsin, or recombinant BSA to replace animal-derived formulations (Figure 1). Animal-free alternatives for BMEs include synthetic polymers, recombinant collagen, and hyaluronic acid-based matrices. Antibodies are another essential research tool that have traditionally been produced in animals. A large-scale study of over 600 commercially available antibodies revealed that more than 50% failed in at least one tested application (Ayoubi et al., 2023), raising concerns about reliability. Notably, the study also demonstrated that recombinant antibodies outperformed both monoclonal and polyclonal counterparts in terms of specificity and reproducibility. Recombinant antibodies are increasingly recommended to enhance reproducibility in research (Kwon, 2024) and are generally considered more animal-friendly, although their development still involves animal use. In contrast, antibodies generated via phage display represent a fully animal-free alternative, as they are selected entirely through *in vitro* processes (Gray et al., 2020; Guliy et al., 2023).

The adoption of serum- and animal-component-free methods depends both on technical feasibility and on a broader cultural shift within the scientific community. As Max Planck famously stated, new scientific paradigms rarely prevail by persuading opponents, but rather by the gradual emergence of a new generation familiar with these ideas from the outset (Planck, 1950). This perspective echoes Thomas Kuhn’s theory of scientific revolutions, which suggests that paradigm shifts often require generational turnover rather than purely rational debate (Kuhn, 1962). Educating the next-generation of scientists in ethical, human-relevant methodologies that avoid the use of animal-derived materials is essential for advancing responsible and reproducible biomedical research. We and others, e.g., (Rafnsdottir et al., 2023), have begun to introduce students to alternatives to animal-derived materials in cell culture.

To further advance these efforts, we have now developed a 1-week lab course focused on cell culture in CDM and the replacement of other animal-derived reagents. Using HeLa cells adapted to CDM, students performed key molecular biology techniques including transfection, RNAi, dual-luciferase assays, qRT-PCR, and Western blotting. Animal-free reagents such as recombinant proteins and phage display antibodies were used in the experiments. While strategies for developing CDMs and employing animal-free reagents are already used in research, albeit with limited dissemination, the key contribution of the present study is their transfer into teaching. Specifically, we describe the conversion of a laboratory course that had been conducted for more than 20 years with FBS and other animal-derived components into one that avoids their use. Successfully implemented at Technische Universität Berlin, the course demonstrates that key experimental workflows can be performed reliably under animal-free conditions. By embedding these methods in higher education, we aim to foster ethically responsible, reproducible, and human-relevant science. All protocols and teaching resources are available for adoption (Supplementary Materials 1, 2).

## 2 Materials and methods

### 2.1 Cell culture

HeLa cells (ACC 57, DSMZ, Braunschweig, Germany) were conventionally cultured in Dulbecco’s Modified Eagle Medium, low glucose (L0064, Biowest, Nuaillé, France), supplemented with 10% FBS (S-10-L, c·c·pro, Oberdorla, Germany), 2 mM L-glutamine (X0,550, Biowest), and 1% non-essential amino acids (NEAA, X0,557, Biowest). The composition of the chemically defined, FBS-free medium is listed in Table 1. Details for the preparation of the medium and cultivation of the HeLa cells in CDM are given in the Standard Operating Procedure (SOP) for HeLa in CDM (Supplementary Material 2). Prepared CDM can be stored at 4 °C for at least 1 month. HeLa cells were directly adapted to CDM without gradual reduction of FBS concentrations over successive passages. Before initiating the switch or passaging, cells were confirmed to be in a highly proliferative state in FBS-supplemented medium. For optimal results and to reduce the influence of residual, intracellular FBS, cells should be cultured in CDM for at least six passages prior to cryopreservation or experimental use.

**TABLE 1:** Composition of the CDM used in this study for culturing HeLa cells.

Component	Concentration	Cat#	Company
DMEM/F-12	1X	L0090	Biowest
Non-essential amino acids	1X	X0557	Biowest
HEPES	15 mM	L1080	Biowest
D-glucose	0.1%	X0550	Sigma-Aldrich, Merck KGaA
L-glutamine	2 mM	25–005-CI	Biowest
Insulin-Transferrin-Selenium	1X	41400045	Gibco, Thermo Fisher Scientific
Hydrocortisone	1 μg/mL	sc-250130	St Cruz Biotechnology
Human epidermal growth factor	10 ng/mL	SRP3027	Sigma-Aldrich, Merck KGaA

Cells were maintained at 37 °C in a humidified incubator with 5% CO_2_. All plasticware, including culture flasks and multiwell plates, was obtained from TPP Techno Plastic Products AG (Trasadingen, Switzerland). Upon reaching 75%–95% confluency, cells were washed with Dulbecco’s phosphate-buffered saline (PBS; L0615, Biowest) and enzymatically detached either using trypsin (L0930, Biowest) or the animal-free reagent TrypLE™ Express (12604021, Gibco™, Thermo Fisher Scientific, Waltham, MA, United States). In the latter case, cells were incubated with TrypLE for 5 min at 37 °C. For cells cultured in CDM, a trypsin inhibitor (TI; 93620-250 MG, Sigma-Aldrich, Merck KGaA, Darmstadt, Germany) was used to neutralize residual enzymatic activity, as CDM lacks the inhibitory proteins naturally present in FBS. In contrast, FBS-containing media inherently inhibit the enzyme due to proteins such as the inter-⍺-trypsin inhibitors, which also contribute to extracellular matrix stability (Chen et al., 1992). To prepare the TrypLE inactivation solution, a 10 mg/mL TI stock solution was diluted to 1 mg/mL in PBS. Aliquots were stored at −20 °C and thawed before use, then equilibrated to room temperature for 5 min. One-twentieth volume of this solution was freshly added to the corresponding volume of base medium and stored at 4 °C until needed. The volume of inactivation solution added per culture vessel is specified in Section 2 of the SOP (Supplementary Material 2). After detachment, cells were centrifuged at 300 *g* for 3 min at room temperature. The resulting cell pellet was resuspended in fresh CDM and seeded into new culture vessels according to the appropriate split ratio.

For the comparison of porcine trypsin and recombinant TrypLE, 1 × 10^5^ cells/well in a volume of 500 µL were seeded into a well of a 24-well plate. After 24 h, cells were detached using either enzyme. At defined time points, the number of detached cells was determined with a Neubauer hemocytometer, and the fraction of detached cells was calculated relative to the maximum detachment observed after 15 min.

Adapted HeLa cells were cryopreserved during the logarithmic growth phase at approximately 95% confluency. Cells were detached as described above, counted with a Neubauer hemocytometer, and centrifuged at 300 *g* for 3 min at room temperature. The resulting pellet was resuspended in a chemically defined freezing medium consisting of 90% CDM and 10% dimethyl sulfoxide (DMSO; D2650, Sigma-Aldrich, Merck KGaA), at a final cell density of 1.5 × 10^6^ cells/mL. Aliquots of 1 mL were dispensed into cryovials (E309, Simport, Carl Roth GmbH + Co. KG, Karlsruhe, Germany) and placed into a Mr. Frosty™ cryo-freezing container (5,100–0,001, Thermo Fisher Scientific) to ensure a controlled cooling rate of −1 °C/min. Vials were stored overnight at −80 °C and transferred the following day into a liquid nitrogen tank for long-term storage. For thawing, cryovials were briefly incubated in a 37 °C water bath for 1–2 min. Subsequently, 1 mL of pre-warmed CDM (37 °C) was slowly added to the cell suspension. Cells were then gently transferred into a T25 culture flask containing 4 mL of pre-warmed CDM. Further details are given in the SOP, Section 2 (Supplementary Material 2).

Osmolality of the media was determined using a freezing point osmometer (Model K7400S, KNAUER Wissenschaftliche Geräte GmbH, Berlin, Germany). Media samples were freshly prepared to prevent alterations in osmolality due to component degradation from freezing or extended storage. Physiological saline served as a reference standard. Each sample was measured in triplicate using 125 µL aliquots per determination.

### 2.2 Phalloidin staining

For phalloidin staining, medium was removed from the cells cultured in 24-well plates, and cells were subsequently rinsed once with PBS and fixed with 200 µL of 4% methanol-free formaldehyde (28908, Thermo Fisher Scientific) for 5 min at room temperature. Residual fixative was removed by two PBS washes. Phalloidin (A12381, Invitrogen, Carlsbad, CA, United States) and DAPI (D1306, Thermo Fisher Scientific) were diluted in PBS to final concentrations of 1:400 and 1:5,000, respectively. For cytoskeletal staining, 200 µL of phalloidin solution was added per well and incubated for 1 h at 37 °C. Subsequently, 100 µL of DAPI solution was added (final concentration: 0.1 μg/mL) and incubated for 30 min at 37 °C in the dark to protect fluorophores from photobleaching. After staining, cells were washed twice with PBS. Fluorescence imaging was performed using a Zeiss Observer Z1 fluorescence microscope (Carl Zeiss AG, Oberkochen, Germany).

### 2.3 Cell viability assay

Metabolic activity was assessed as an indicator of cell viability using the XTT assay, a colorimetric method based on the mitochondrial-dependent reduction of the tetrazolium salt XTT (2,3-bis-(2-methoxy-4-nitro-5-sulfophenyl)-2H-tetrazolium-5-carboxanilide) by cellular dehydrogenases. The XTT reagent was prepared by combining XTT (J61726. MD, Thermo Fisher Scientific, 1 mg/mL) with phenazine methosulfate (PMS, 3.83 mg/mL; P9625, Sigma-Aldrich, Merck KGaA) in a 1:500 ratio. For each sample, 0.5 vol. of the freshly prepared XTT/PMS solution was added to the medium in wells of a 48-well plate and incubated for 4 h at 37 °C in a humidified atmosphere containing 5% CO_2_. Following incubation, 100 µL of the supernatant was transferred to a 96-well plate, and absorbance was measured at 450 nm with a reference wavelength of 620 nm using a microplate reader (Sunrise, Tecan Group Ltd., Männedorf, Switzerland). To account for background signal, a negative control was included by treating cells with 70% ethanol for 10 min prior to addition of the XTT/PMS solution.

### 2.4 Design and transfection of small interfering RNAs (siRNAs)

The siRNA targeting the Golgi Protein 73 (GP73) was synthesized by Microsynth (Microsynth AG, Balgach, Switzerland) based on a previously published sequence (Liu et al., 2016), and was used in all subsequent gene-silencing experiments. A non-targeting control siRNA, exhibiting no sequence homology to human or viral genomes, served as the negative control. Furthermore, a fluorescently labeled siRNA was used to evaluate the transfection efficiency. The sequences of siRNAs used in this study are provided in Table 2; in the case of the GP73-targeting siRNA, the antisense strand is complementary to the target mRNA.

**TABLE 2:** List of siRNA oligonucleotides used for functional knockdown and gene silencing.

siRNA	Nucleotide sequence [5’ – 3’]		Application
siGP73	Sense	CAA​GCU​GUA​CCA​GGA​CGA​A [dTdT]	siRNA against GP73
	Antisense	UUC​GUC​CUG​GUA​CAG​CUU​G [dTdT]	
siCon	Sense	ACU​ACC​GUU​GUU​AUA​GGU​G [dTdT]	scrambled siRNA used as negative control
	Antisense	UGA​UGG​CAA​CAA​UAU​CCA​C [dTdT]	
siCy3	Sense	UUC​UCC​GAA​CGU​GUC​ACG​U [dTdT]	siRNA conjugated with fluorophore Cy3 at the 5′ end of the sense strand
	Antisense	ACG​UGA​CAC​GUU​CGG​AGA​A [dTdT]	

Transfection of siRNAs with Lipofectamine 2000 (11668027, Invitrogen) and the K4 transfection reagent (T080-1.0, Biontex Laboratories GmbH, Munich, Germany) were carried out according to the manufacturer’s instructions. The transfection efficiency was evaluated by quantifying the signal from Cy3-labelled siRNAs (siCy3). The fluorescence micrographs were analyzed in ImageJ (FIJI, v1.53) by counting cells with positive signal. The proportion of positively transfected cells was then compared between the two reagents.

### 2.5 Assessment of silencing efficiency via dual-luciferase reporter assay

The silencing efficiency of siGP73 was initially evaluated using a dual-luciferase reporter assay. For this purpose, a reporter construct based on the psiCHECK™-2 vector (C8021, Promega, Madison, WI, United States) was used, in which the Renilla luciferase (RLuc) gene is fused to the GP73 target sequence. This vector also expresses Firefly luciferase (FLuc) from a separate promoter, serving as an internal control to normalize for transfection efficiency and cell viability. To generate the reporter plasmid, the GP73 target sequence was synthesized with appropriate overhangs, annealed, and cloned downstream of the RLuc gene using the XhoI and NotI restriction sites. The recombinant plasmid was propagated in *E. coli*, and purified using the NucleoSpin^®^ Plasmid EasyPure Kit (740727, Macherey-Nagel, Düren, Germany) according to the manufacturer’s protocol. HeLa cells (1 × 10^5^ cells/well) were seeded in 24-well plates and co-transfected 24 h later with 500 ng of the reporter plasmid and 0.1, 1, or 10 nM siRNA (siGP73 or non-targeting control siRNA, siCon) in Opti-MEM (11058, Gibco™, Thermo Fisher Scientific). Transfections were performed using the K4 transfection reagent, following the manufacturer’s instructions. Forty-eight hours post-transfection, luciferase activities were measured using a non-commercial dual-luciferase assay described by Hampf and Gossen (Hampf and Gossen, 2006), providing a cost-effective and reliable alternative to commercial kits. The assay allows sequential quantification of Firefly (*Photinus pyralis*) and Renilla (*Renilla reniformis*) luciferase activity from the same sample. Firefly luminescence was measured first, followed by quenching and subsequent measurement of RLuc activity. RLuc activity, reflecting the effect of siRNA on the GP73 target sequence, was normalized to FLuc activity. The resulting RLuc/FLuc ratio was used to assess knockdown efficiency and to improve the accuracy and reproducibility of the results.

### 2.6 Quantification of GP73 mRNA expression by qRT-PCR

RNAi-mediated knockdown of GP73 was quantified by qRT-PCR. To this end, HeLa cells (1 × 10^5^ cells per well) were seeded in 24-well plates and transfected after 24 h with 10, 50, or 80 nM of either siGP73 or a non-targeting control siRNA (siCon), using K4 transfection reagent according to the manufacturer’s protocol. Forty-eight hours post transfection, total RNA was extracted directly from cultured cells using RLT buffer (79216, Qiagen, Hilden, Germany) and the RNeasy Mini Kit (74104, Qiagen), following the provided instructions. RNA concentration and purity were assessed using a NanoDrop™ 2000 spectrophotometer (Thermo Fisher Scientific). To eliminate potential genomic DNA contamination, samples were treated with RQ1 RNase-Free DNase (M6101, Promega). Complementary DNA (cDNA) synthesis was performed using the RevertAid H Minus First Strand cDNA Synthesis Kit (1,632, Thermo Fisher Scientific). Quantitative reverse transcription PCR (qRT-PCR) was conducted on a CFX Opus 96 Real-Time PCR System (Bio-Rad Laboratories GmbH, Feldkirchen, Germany) using SsoFast EvaGreen Supermix (1725204, Bio-Rad). Reactions were set up in a 20 µL volume with the following thermal cycling conditions: initial denaturation at 95 °C for 30 s, followed by 40 cycles of 95 °C for 5 s and 60 °C for 5 s. Melt curve analysis (65 °C–95 °C) confirmed specificity of amplification. Gene expression levels of GP73 were normalized to 18S rRNA, and relative expression was calculated using the 2^−ΔΔCT^ method (Livak and Schmittgen, 2001), with siCon-transfected cells serving as the reference. Primer sequences for GP73 and 18S rRNA are listed in Table 3.

**TABLE 3:** Nucleotide sequences of qPCR primers used in this study.

Target	Primer	Nucleotide sequence [5’ – 3’]	Application
GP73	Forward	CAG​CGC​TGA​TTT​TGA​GAT​GAC	qPCR amplification
	Reverse	ATG​ATC​CGT​GTC​TGG​AGG​TC	
18S rRNA	Forward	CGC​GGT​TCT​ATT​TTG​TTG​GT	qPCR normalization control
	Reverse	AGT​CGG​CAT​CGT​TTA​TGG​TC	

### 2.7 Western blot analysis of GP73 expression

Western blotting was performed to evaluate GP73 protein levels following siRNA-mediated knockdown. HeLa cells (2 × 10^5^ cells per well) were seeded in 12-well plates and cultured in medium containing FBS or CDM as indicated. Twenty-four hours post seeding, cells were transfected with 10, 50, or 80 nM of either siGP73 or siCon using K4 transfection reagent (Biontex Laboratories GmbH), according to the manufacturer’s instructions. Forty-eight hours post-transfection, cells were lysed in RIPA buffer (89900, Invitrogen), and total protein concentrations were determined using the Bicinchoninic Acid (BCA) Protein Assay Kit (23227, Thermo Fisher Scientific). Equal amounts of protein were mixed with NuPAGE LDS Sample Buffer (NP0007, Invitrogen), supplemented with 50 mM dithiothreitol (DTT, 6,908.1, Carl Roth GmbH + Co. KG), and denatured by heating at 95 °C for 5 min. Samples were then cooled on ice. Proteins were separated by 10% SDS-PAGE at 120 V and transferred onto PVDF membranes using a semi-dry blotting system at 70 mA per blot for 70 min. Membranes were blocked with 5% soy milk (Allos Soy Milk Barista, Allos Hof-Manufaktur GmbH, Bremen, Germany) in TBS-T for 45 min at room temperature. The blots were incubated overnight at 4 °C with monoclonal mouse anti-GP73 primary antibody (1:200, sc-393372, Santa Cruz Biotechnology, Heidelberg, Germany) diluted in TBS-T. After three washes with TBS-T (5 min each), membranes were incubated for 1 h at room temperature with a recombinant HRP-conjugated secondary antibody (1:5,000, sc-542731, Santa Cruz Biotechnology), followed by three additional washes in TBS-T. Protein detection was performed using the ECL Western blotting Substrate (32209, Pierce, Thermo Fisher Scientific), and chemiluminescence was visualized using a ChemiDoc MP Imaging System (Bio-Rad Laboratories GmbH).

Actin was used as a loading control. Two antibody-based detection strategies were employed, as described in the main text. In the conventional approach, a monoclonal mouse anti-actin antibody (A5441, 1:5,000, Sigma-Aldrich, St. Louis, MO, United States) was incubated with the membrane for 24 h at 4 °C, followed by a 1 h incubation at room temperature with an HRP-conjugated goat anti-mouse secondary antibody (31430, 1:10,000, Thermo Scientific, Pierce™). As an animal-free alternative, an HRP-conjugated phage display-derived anti-actin antibody (HCA147P, Bio-Rad Laboratories GmbH) was applied for 24 h at 4 °C. All antibodies were diluted in TBS-T. Protein detection was performed as described above. Western blots were quantitatively evaluated with the Image Lab software, Version 5.0 build 18 (Bio-Rad Laboratories GmbH).

### 2.8 Statistical analysis

All experiments were conducted in triplicate and independently repeated at least three times. Data are presented as mean ± standard deviation (SD), unless otherwise specified. Statistical analyses were performed using GraphPad Prism software Version 8.0.2. Statistical significance was defined as an adjusted p-value <0.05. The following thresholds were used to indicate significance: *p ≤ 0.05, **p ≤ 0.01, ***p ≤ 0.001, and ****p ≤ 0.0001.

### 2.9 Ethical statement

The materials used in this study are not entirely free of animal-derived components, primarily for scientific reasons: the study aimed to compare data from experiments using established protocols that include animal-derived materials with those employing animal-free alternatives. Nonetheless, we are committed to continuously improving our sourcing and production processes to align with our stringent ethical standards and evolving societal expectations. Additionally, our laboratory actively supports the United Nations Sustainable Development Goals (SDGs) and has implemented comprehensive sustainability measures across all research and teaching activities. These efforts have been recognized by certification from “My Green Lab” at the highest sustainability level, “Green”. Sustainability measures in biomedical research can also be introduced in the laboratory course.

## 3 Results

**FIGURE 2:**
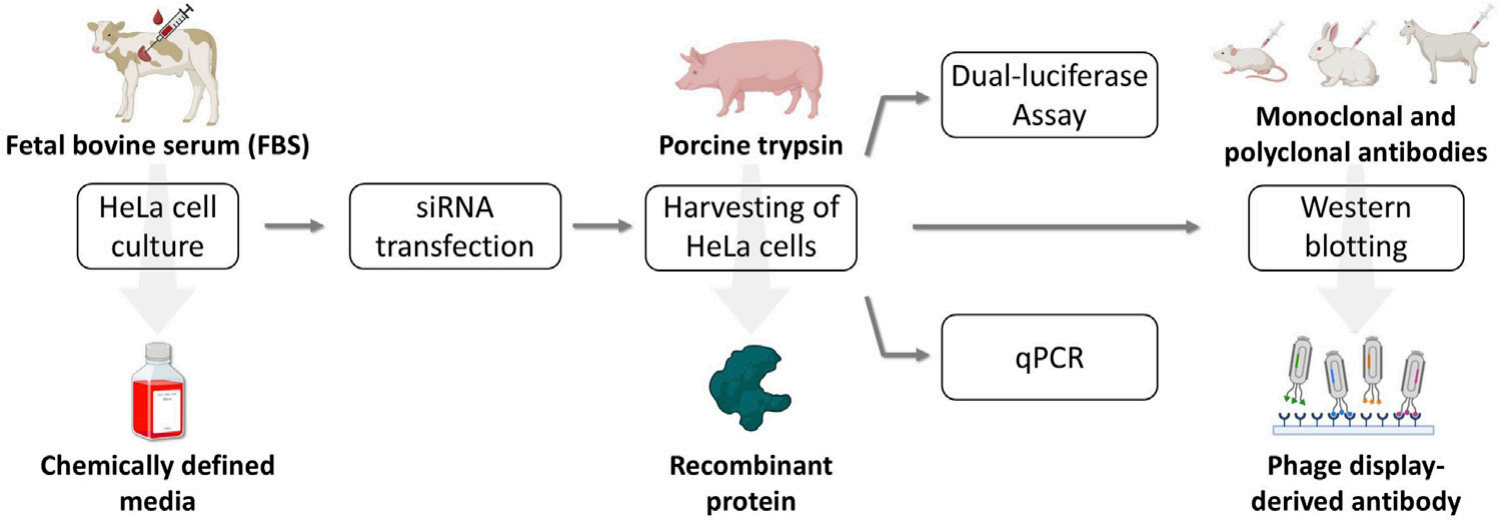
Flow diagram of the laboratory course to be developed, designed to replace animal-derived components. HeLa cell cultivation was switched from FBS-supplemented medium to a CDM. For cell detachment, recombinant TrypLE was used instead of porcine trypsin. A certified animal-free qRT-PCR kit was employed, and for Western blotting of actin the conventional combination of a primary monoclonal and a secondary polyclonal antibody was replaced with a phage display-derived antibody. Created with BioRender.com.

To prepare the practical course, suitable conditions were first established. An overview of the laboratory course to be developed, highlighting the replacement of animal-derived materials, is presented in Figure 2. HeLa cells were adapted to FBS-free cultivation in a synthetic medium, followed by analyses of proliferation, metabolic activity as an indicator of cell viability, and filamentous actin (F-actin) structures. Subsequently, trypsinization with recombinant TrypLE and Western blotting for GP73 and actin were optimized using antibody variants that significantly reduce or eliminate animal use. After method validation, siRNA-mediated knockdown was performed under both FBS-containing and fully synthetic conditions. The results in CDM were then verified during the practical course by four student groups.

### 3.1 Cultivation of HeLa cells under FBS-Free culture conditions

As an initial step, HeLa cells previously maintained in FBS-supplemented medium were adapted to CDM. Cells were thawed and cultured in standard medium containing 10% FBS, designated as passage 0. Following recovery and stabilization, cells were transferred to CDM at passage 1. To ensure optimal growth conditions, the medium was refreshed every 2–3 days, and passaging was performed only after cells reached at least 80% confluency to minimize adaptation-related stress. This protocol was maintained for a minimum of six consecutive passages in CDM. Osmolality measurements of FBS-containing medium and CDM showed no significant differences, with both falling within the physiological range of 260–310 mOsmol/kg, consistent with established standards for mammalian cell culture (Supplementary Figure 1) (Ozturk and Palsson, 1991). By the end of this stage, HeLa cells demonstrated stable proliferation and morphology comparable to those maintained in FBS-supplemented medium, indicating successful adaptation to the CDM.

**FIGURE 3:**
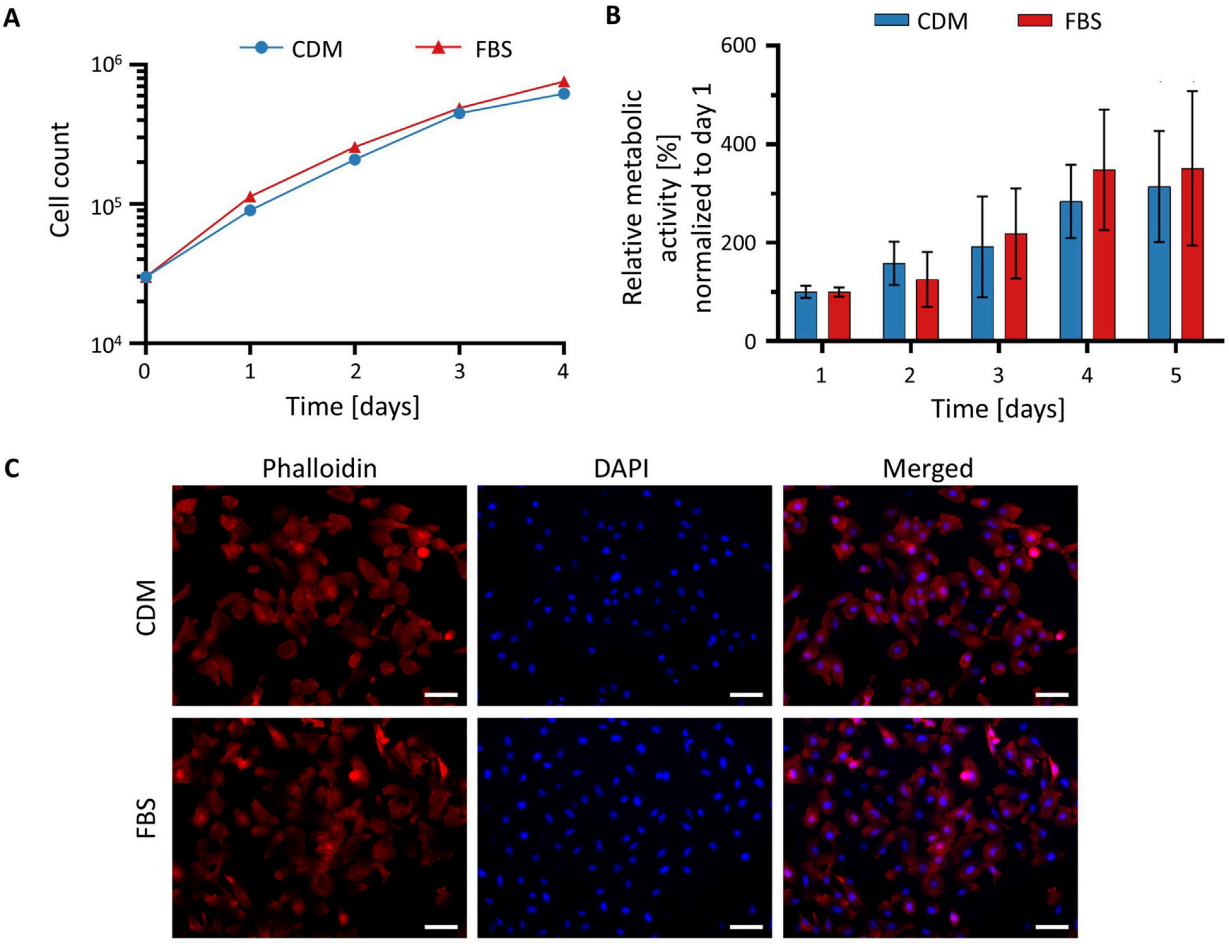
Cultivation of HeLa cells in CDM. **(A)** Growth curves of HeLa cells cultured in CDM (blue circles) or FBS-supplemented medium (red triangles) over a 4-day period. Proliferation was assessed by direct cell counting at the indicated time points using cultures at passage six or higher. **(B)** Cell viability assessed by XTT assays. Metabolic activity of cells cultured in medium with FBS (red) or CDM (blue) was quantified by measuring absorbance at 450 nm. Higher absorbance indicates greater metabolic activity and cell viability. All values were normalized to the value for day 1. Values in **(A,B)** represent mean ± SD (*n* = 3). Statistical analysis was performed using an unpaired *t*-test; *p* < 0.05 was considered statistically significant. **(C)** Phalloidin and DAPI staining of HeLa cells cultured in conventional medium containing FBS and CDM. Cells were fixed and stained with phalloidin to visualize F-actin (red) and DAPI to label nuclei (blue). Scale bar: 100 µm.

To determine whether CDM supports HeLa cell proliferation comparably to standard FBS-containing medium, 30,000 cells per well were seeded into a 24-well plate on day 0, and changes in cell number were monitored over a 4-day period. As shown in Figure 3A, the growth curves of cells cultured in CDM and in medium supplemented with 10% FBS were nearly identical across all time points. Quantitative analysis of cell counts revealed no statistically significant differences in proliferation between the two conditions, indicating that CDM supports HeLa cell growth to a similar extent as conventional FBS-supplemented medium. The doubling time was approximately 12 h on the first day after seeding and, according to the method described in (29), was calculated to be approximately 29 h under each condition thereafter.

To assess the metabolic activity as a measure for cell viability, an XTT assay was performed. On day 0 of the experiment, 0.5 × 10^4^ cells/well in a volume of 100 µL were seeded into a well of a 96-well plate and metabolic activity was measured daily starting on day 1. Absorbance measurements at 450 nm, which reflect mitochondrial metabolic activity, were comparable between both culture conditions (Figure 3B). An unpaired t-test confirmed that the differences were not statistically significant, suggesting that CDM does not compromise cell viability. Collectively, these results demonstrate that CDM provides an effective alternative to FBS-containing medium, supporting HeLa cell viability and proliferation at equivalent levels.

To evaluate potential intracellular differences between HeLa cells cultured in conventional FBS-containing medium and CDM, cells were stained with phalloidin to visualize F-actin and DAPI to label nuclei. As shown in Figure 3C, HeLa cells displayed characteristic well-organized actin cytoskeletons and evenly distributed nuclei. Cells cultured in FBS-containing medium appeared slightly more elongated and formed denser clusters, displaying prominent actin-rich extensions suggestive of enhanced spreading and intercellular interaction. In contrast, cells maintained in CDM showed a somewhat more heterogeneous morphology, with a tendency toward a more rounded or polygonal shape. Nonetheless, F-actin organization remained well-defined, and nuclear morphology was comparable under both culture conditions. Upon closer examination, a few multinucleated cells were observed in cultures maintained in the chemically defined medium. DAPI staining demonstrated similar nuclear density across conditions, indicating that the observed differences were not due to variations in cell number or proliferation. These results confirm that HeLa cells can be successfully maintained and imaged in a chemically defined, animal-free medium, supporting its use for research and educational applications.

### 3.2 Efficiency of cell detachment using trypsin and TrypLE

**FIGURE 4:**
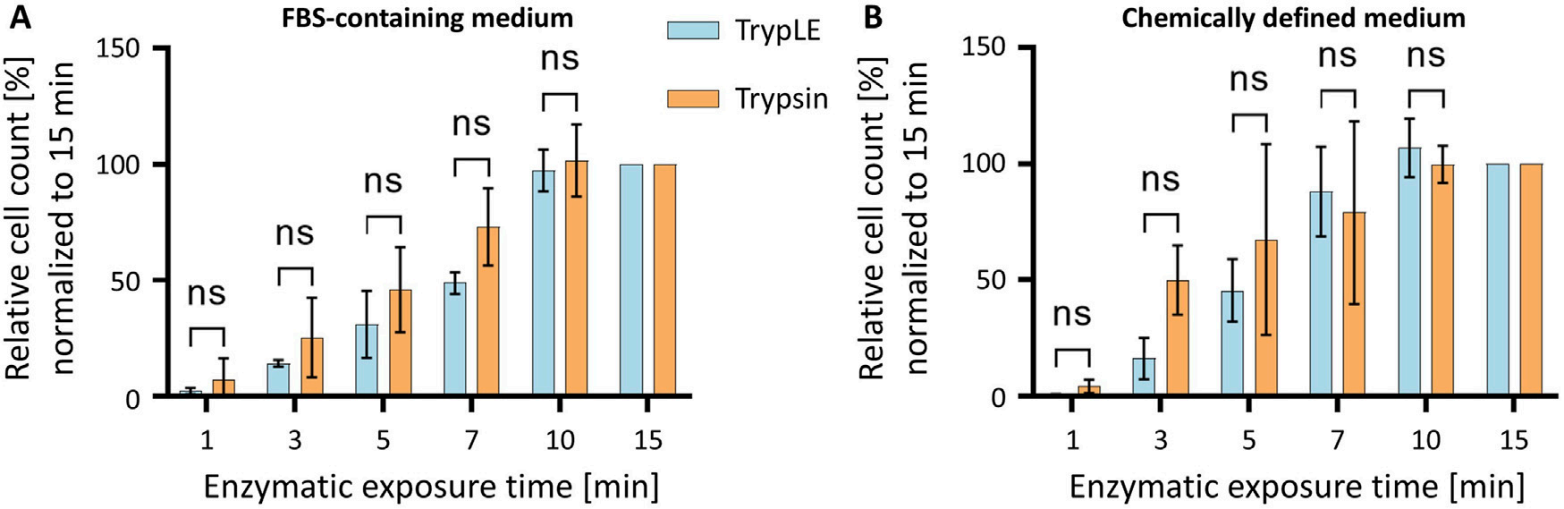
Time-dependent detachment of HeLa cells cultured in different media using Trypsin or TrypLE. **(A)** HeLa cells cultured in FBS-supplemented medium and **(B)** HeLa cells cultured in CDM were treated with TrypLE (blue) or Trypsin (orange) for up to 15 min. The number of detached cells was normalized to the maximum value observed. Data are presented as mean ± SD (*n* = 3).

Porcine trypsin is widely used for cell detachment in mammalian cell culture. To assess the performance of a recombinant, animal-free alternative, HeLa cells maintained in either FBS-supplemented medium or CDM were detached using one of the two enzymes. In both, FBS-containing conditions and the CDM comparable results were obtained (Figure 4). Both enzymes exhibited a time-dependent increase in the number of detached cells. Trypsin induced a slightly faster detachment at early time points compared to TrypLE. By 10 min, however, both enzymes reached maximal detachment, with no further increase observed at 15 min. These results demonstrate that both Trypsin and TrypLE are effective for detaching HeLa cells, regardless of the culture medium, i.e., TrypLE provides a viable animal-free alternative for routine cell passaging.

### 3.3 RNA interference-mediated knockdown of the golgi phosphoprotein 73 (GP73) in HeLa cells cultured in FBS-Containing and chemically defined medium

To assess RNAi efficiency in both FBS-containing media and CDM, a series of experiments was conducted. Transfection efficiency was first evaluated by fluorescence microscopy using Cy3-labelled siRNA (siCy3) and two different transfection reagents. GP73 knockdown in HeLa cells was then analyzed by dual-luciferase assay, qRT-PCR, and Western blotting.

**FIGURE 5:**
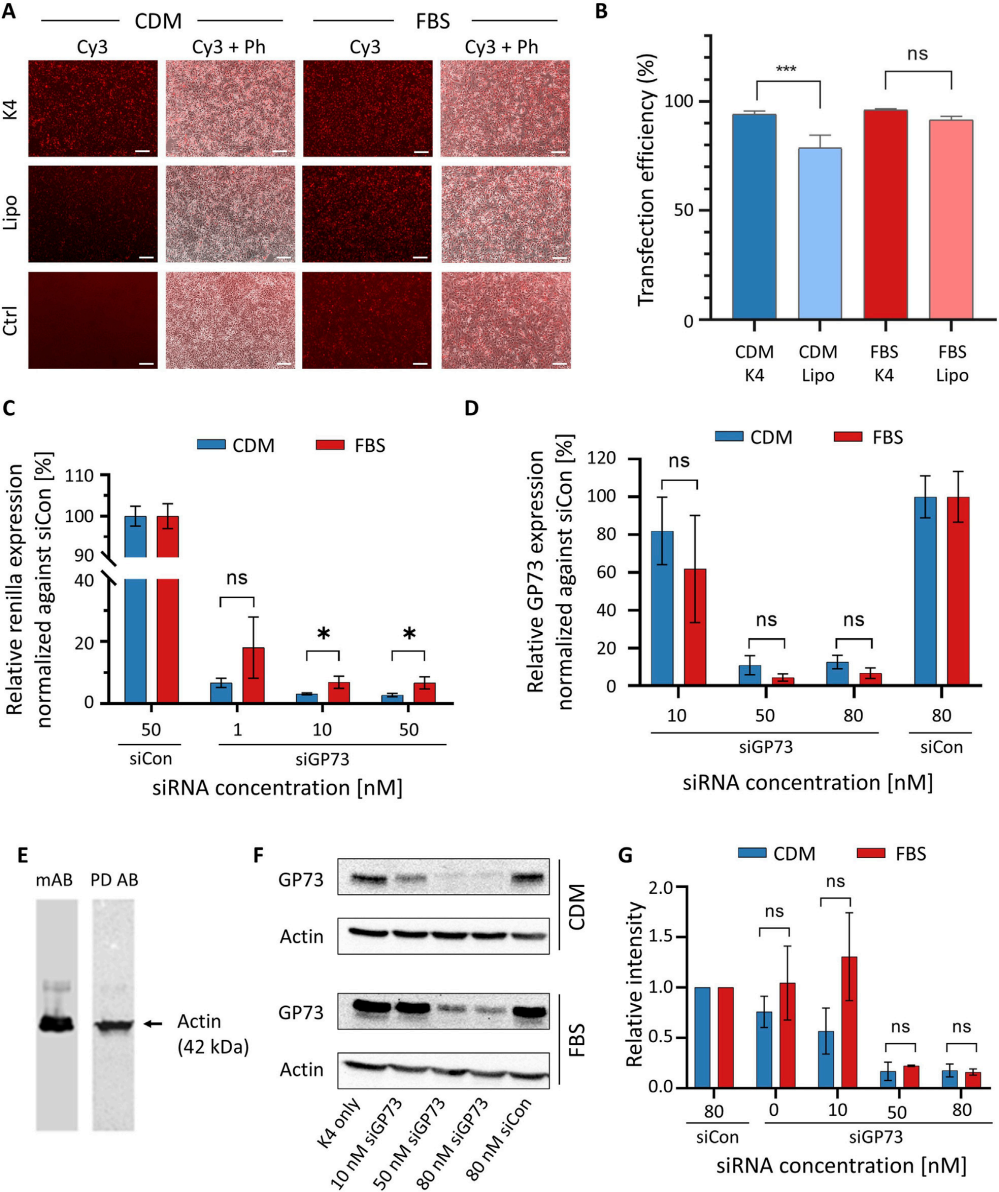
RNAi experiments in HeLa cells cultured in FBS-supplemented medium and CDM. **(A)** Investigation of transfection efficiency of siRNA. HeLa cells were transfected with 40 nM Cy3-labeled siRNA (siCy3) for 48 h using either K4 or Lipofectamine 2000 (Lipo) as transfection reagents in FBS-containing medium or CDM. A control condition (Ctrl) in which siRNA was applied without transfection reagent was included. Fluorescence microscopy was used to visualize Cy3 signal as an indicator of transfection efficiency. Representative fluorescence (Cy3) and merged phase contrast (Ph) images are shown. Scale bar: 200 μm. **(Β)** Quantitative analysis of transfection with K4 and Lipofectamine 2000 in cells cultured in CDM and FBS-containing medium, respectively. Data represent results from three independent experiments and were analyzed using ImageJ (FIJI). **(C)** Dual-luciferase reporter assay demonstrating siGP73-mediated gene silencing in HeLa cells. HeLa cells cultured in CDM (blue) or FBS-containing medium (red) were co-transfected with a reporter plasmid expressing RLuc fused to the GP73 target sequence and either siGP73 (1, 10, or 50 nM) or a non-targeting siRNA control (siCon, 50 nM). RLuc activity, representing GP73-targeted silencing, was normalized to Firefly luciferase as an internal control. **(D)** Quantification of endogenous GP73 mRNA expression by qRT-PCR following transfection with siGP73 (10, 50, or 80 nM) or non-targeting control siRNA (siCon, 80 nM). Expression levels were normalized to 18S rRNA and are shown relative to each siCon. Data represent mean ± SD (n = 3). **(E)** Western blots for actin with cell extracts from cultures in CDM. The experiment was either carried out with a monoclonal antibody (mAb) followed by a secondary polyclonal goat-anti-mouse antibody or with a HRP-conjugated phage display-derived antibody (PD Ab). **(F)** Representative Western blot analysis of GP73 protein expression after transfection with increasing concentrations of siGP73 or 80 nM siCon. The K4-only group (transfection reagent without siRNA) served as an additional control. Actin was used as a loading control. **(G)** Quantitative analysis of Western blots for GP73 knockdown based on three independent experiments.

HeLa cells maintained in either CDM or conventional FBS-supplemented medium were transfected with Cy3-labeled siRNA using either the K4 transfection reagent or Lipofectamine 2000. Both transfection reagents are based on cationic lipids, although their exact compositions are proprietary and not disclosed by the manufacturers. The purpose of the experiment is to demonstrate to students how to select an appropriate transfection reagent, a routine procedure in cell culture practice. Both K4 and Lipofectamine enabled robust Cy3 fluorescence, confirming successful siRNA delivery in both culture conditions (Figure 5A). While overall transfection efficiency appeared comparable, quantitative analysis showed that K4 performed slightly but statistically significantly better than Lipofectamine in CDM-cultured cells (Figure 5B). Merged fluorescence and phase-contrast images revealed consistent cell morphology and confluency across all conditions, indicating that culture in CDM did not adversely affect transfection efficiency or cell viability. In the control condition, where Cy3-labeled siRNA was applied without a transfection reagent, no fluorescence signal was detected in CDM. Interestingly, a weak signal was observed under FBS-containing conditions, potentially reflecting nonspecific uptake or background fluorescence associated with serum components. These results demonstrate that CDM is fully compatible with siRNA transfection in HeLa cells and that the K4 reagent represents a cost-effective and efficient alternative to Lipofectamine, particularly for culture in CDM.

To study the silencing efficiency of siGP73 via a dual-luciferase reporter assay in HeLa cells cultured in either CDM or FBS-supplemented medium, cells were co-transfected with a psiCHECK™-2-based reporter plasmid encoding Renilla luciferase (RLuc) fused to a fragment of the GP73 sequence, along with siGP73 at concentrations of 1, 10, or 50 nM, or 50 nM of siCon. A concentration-dependent decrease in normalized RLuc activity compared to siCon was induced (Figure 5C). A moderate silencing effect was observed at 1 nM, while 10 and 50 nM siGP73 achieved robust knockdown. These results demonstrate effective and specific GP73 silencing under both FBS-free and FBS-containing conditions.

Silencing efficiency of siGP73 on endogenous GP73 expression was assessed in HeLa cells transfected with increasing concentrations of siGP73 (10, 50, or 80 nM) or 80 nM of the non-targeting control siRNA (siCon), and cultured in either CDM or FBS-supplemented medium. Knockdown was quantified by qRT-PCR using the SsoFast™ EvaGreen^®^ Supermix (Bio-Rad), chosen for its animal-free certification, unlike similar products that may contain animal-derived materials such as BSA. The qRT-PCR analysis revealed a concentration-dependent reduction in GP73 mRNA levels under both conditions (Figure 5D). A modest decrease was observed at 10 nM siGP73, while 50 and 80 nM siGP73 resulted in robust knockdown. In contrast, siCon-treated cells maintained high GP73 expression.

To validate the qRT-PCR findings at the protein level, Western blot analysis was performed. Actin, a widely used internal standard, served as a loading control to confirm equal protein loading. Following standard molecular biology protocols, we have traditionally used a monoclonal mouse anti-actin antibody as the primary antibody, followed by a polyclonal goat anti-mouse secondary antibody, in both research (Ziersch et al., 2024) and teaching settings. As this approach raises ethical concerns due to its reliance on animal-derived components, we evaluated a phage display-derived anti-actin antibody conjugated to horseradish peroxidase (HRP) as an animal-free alternative. Both detection systems produced a single, specific band (Figure 5E), demonstrating comparable performance. The phage display-derived antibody not only eliminates the need for animal-derived antibodies but also streamlines the protocol by removing the secondary antibody incubation step.

Since no phage display-derived antibodies were available for the target protein GP73, we used a monoclonal antibody in combination with a recombinant secondary antibody. The use of different antibody types within the same experimental framework fosters discussion on ethical considerations and highlights the practical advantages of animal-free alternatives. To assess GP73 silencing, HeLa cells were transfected with increasing concentrations of siGP73. Substantial knockdown was observed at 50 and 80 nM, whereas cells transfected with siCon showed no significant change in GP73 expression (Figure 5F). Actin detection using the phage display-derived antibody confirmed equal protein loading across conditions. Densitometric analysis of three independent Western blots, despite the relatively high variability typically associated with quantitative evaluation of Western blotting, demonstrated approximately 80% knockdown of the target gene at 50 and 80 nM siRNA (Figure 5G). Importantly, no significant differences were observed between experiments performed in FBS-containing standard medium and CDM.

### 3.4 Practical laboratory course demonstrates successful implementation of animal-free experimental workflow

The FBS-free CDM used in this study was developed and validated by the research group, based on extensive experience with cell culture protocols traditionally reliant on FBS. Following successful protocol development and reformulation of the laboratory script, it was implemented in a practical training module for graduate Biotechnology students at Technische Universität Berlin. During the course, students were introduced to the ethical, scientific, and technical implications of using animal-derived components in modern cell culture. They were divided into four groups (of three students each), and performed all experiments using cells cultured in CDM exclusively.

**FIGURE 6:**
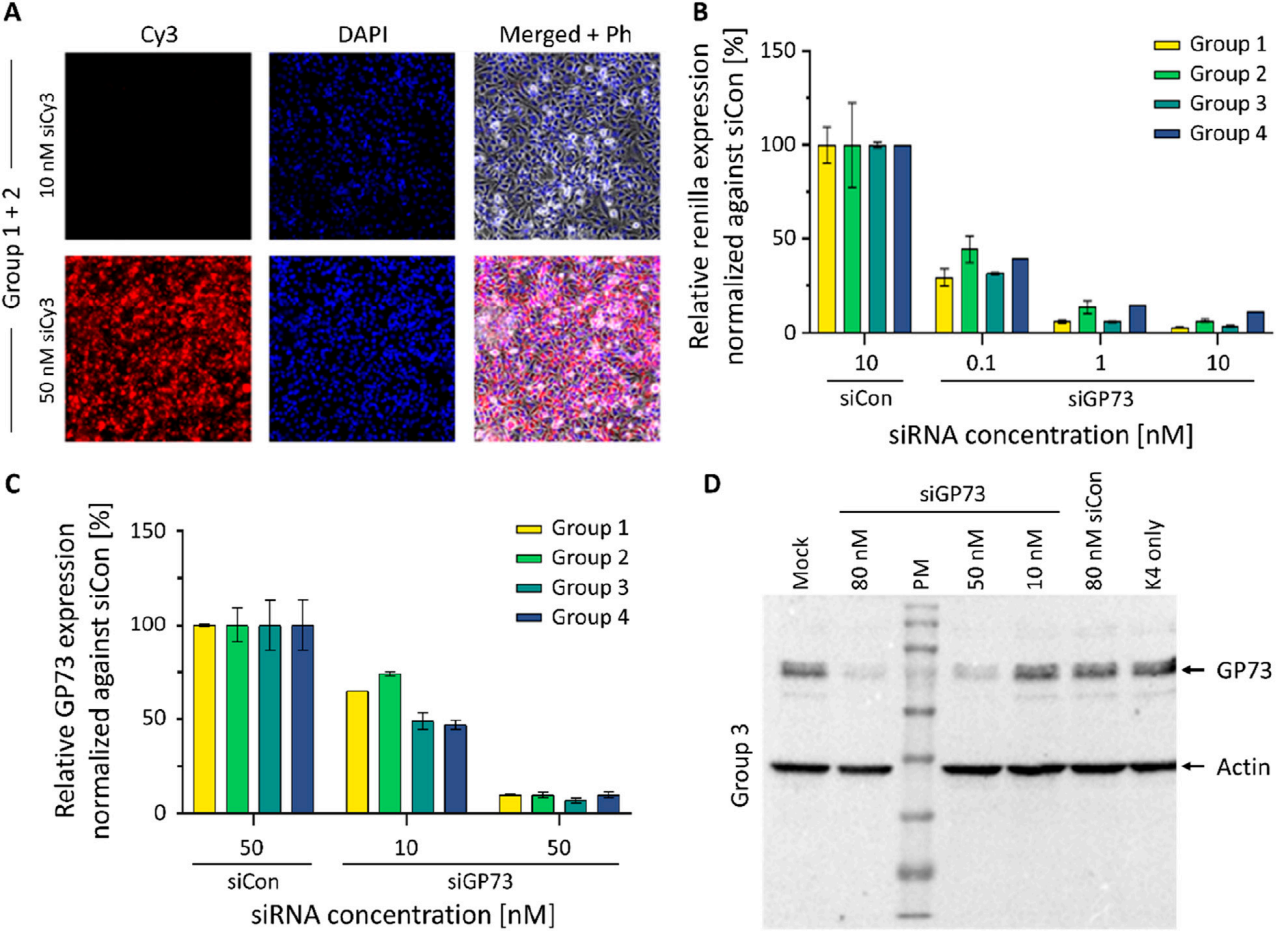
Results of the student laboratory course. **(A)** Experiment 1: Transfection of a Cy3 modified siRNA. Cy3-labeled siRNAs were transfected into HeLa cells cultured in CDM at concentrations of 10 and 50 nM. Fluorescence microscopy images of formaldehyde-fixed cells were taken 24 h post-transfection. **(B)** Experiment 2: Dual Luciferase Assay (DLA). HeLa cells cultured in CDM were transfected with an siRNA targeting GP73 (siGP73) at increasing concentrations as indicated. The relative reporter activity was compared to a non-targeting control siRNA (siCon, 10 nM). Renilla luciferase activity, linked to the siGP73 target sequence, was normalized to Firefly luciferase as an internal control. Reduced Renilla/Firefly ratios indicate effective siRNA-mediated silencing. **(C)** Experiment 3: Quantitative RT-PCR. HeLa cells were transfected with GP73-targeting siRNA (siGP73) at 10 nM and 50 nM or with non-targeting control siRNA (siCon, 50 nM). GP73 mRNA expression levels were measured 48 h post-transfection and normalized to the siCon group. Bars represent the mean of technical replicates from four independent experiments; error bars indicate standard deviation (SD). **(D)** Western blot analysis of GP73 expression following siRNA transfection. HeLa cells were transfected using K4 transfection reagent with a GP73-targeting siRNA (siGP73) at 10, 50, or 80 nM. Controls included a non-targeting control siRNA (siCon, 80 nM), a reagent-only control (K4 without siRNA), and an untransfected mock control (medium only). Protein lysates were collected 48 h post-transfection. GP73 protein expression was detected via Western blotting; β-actin was used as a loading control. PM = protein marker.

Students evaluated transfection efficiency by comparing different reagents and varying concentrations of a Cy3-labeled siRNA. Fluorescence microscopy confirmed efficient uptake of the siRNA in HeLa cells cultured in CDM (Figure 6A). Functional activity of the siRNA was subsequently demonstrated using a dual-luciferase reporter assay (Figure 6B). Concentration-dependent gene silencing of the endogenously expressed target was further validated by qRT-PCR (Figure 6C) and Western blot analysis (Figure 6D). Results generated during the course were benchmarked against archived data obtained using FBS-containing medium, confirming comparable performance. A second implementation of the course further supported the reproducibility and robustness of the CDM-based protocols (data not shown). The course received highly positive overall evaluations, with students particularly emphasizing the methodological knowledge they had gained. Some criticism concerned errors in the script, which were subsequently corrected, as well as waiting times resulting from the limited number of workstations in our student laboratory. A full transcript of the survey including descriptive statistics is given as Supplementary Material 3. The full experimental script and detailed instructions used in the laboratory course are provided in Supplementary Material 1.

## 4 Discussion

The present study illustrates that FBS-free cell culture using CDM, in combination with non-animal alternatives for downstream applications, can be effectively implemented in an educational setting. Since the advent of immortalized human cell lines, FBS has become a standard supplement in cell culture. However, its continued use is increasingly questioned due to ethical concerns and scientific limitations, such as batch variability and undefined composition (Weber et al., 2021). CDMs offer a promising alternative, enhancing reproducibility, eliminating xenogeneic components, and improving physiological relevance (Subbiahanadar Chelladurai et al., 2021). This shift is particularly relevant for both academic and industrial research, including toxicity testing (Pfeifer et al., 2024).

Several strategies are available for transitioning away from FBS, including the use of commercially available media, the development of cell line–specific formulations (Wijerathna-Yapa et al., 2025), and the application of universal FBS-free media suitable for multiple cell types (Rafnsdottir et al., 2023; Weber et al., 2024). However, commercially available media are often prohibitively expensive and their undisclosed composition hampers reproducibility, which stands in contrast to the principles of open science. Universal media facilitate broader adoption but are also costly; therefore, in contexts such as teaching or large-scale production of biotechnological products, the development of cell line–specific media may represent a more practical alternative. Building on our previous development of a CDM for HuH-7 cells (Ali et al., 2024a), we subsequently optimized a CDM tailored for HeLa cells. Cells proliferated continuously in the developed CDM with a doubling time of approximately 29 h, which was nearly identical to that observed in conventional FBS-containing medium (Figure 3A). Malakpour-Permlid reported that HeLa cells can also be cultured in the same universal medium with a doubling time of 57.5 h (Malakpour-Permlid et al., 2025). By contrast, the reported doubling time of 22–32 h in standard medium is comparable to that observed in our study.

HeLa cells are associated with a complex ethical history, thoughtfully explored in Rebecca Skloot’s book *The Immortal Life of Henrietta Lacks* (Skloot, 2010). In addition, these cells are associated with certain scientific concerns, including genetic instability and the potential for cross-contamination with other cell lines. Nevertheless, they were deliberately selected for the present laboratory course, as they remain a widely used and biologically well-characterized model system. Their inclusion also provides an opportunity to engage students in broader discussions on patient consent and research ethics within biomedical education and practice.

Despite the availability of alternatives, FBS remains widely used. Cost is often cited, though price differences have narrowed. To illustrate this, we calculated the cost of a 500 mL bottle of the CDM for HeLa cells developed here based on current pricing at Technische Universität Berlin as of May 2025 (see Supplementary Material 4). While the FBS-containing medium remains less expensive, the difference is modest: €30.65 (plus tax) for the standard medium *versus* €53.28 (plus tax) for the CDM. It should be noted that we obtain FBS from a supplier offering high-quality serum at a comparatively low price. Other suppliers charge substantially higher prices for FBS, which would reduce the cost difference between conventional medium and CDM. In addition to cost, physiological changes associated with CDM adaptation, such as slower proliferation, also contribute to resistance. Yet, for many applications – including reporter assays, teaching, or virus production – these differences are minimal. Moreover, FBS promotes unphysiologically high proliferation, raising questions about its relevance.

It is important to note that HeLa cells, even when cultured under standardized conditions with identical FBS supplementation, can exhibit variability in growth characteristics depending on their source and laboratory handling. For instance, HeLa cells obtained from different commercial vendors and propagated in separate laboratories often show divergent doubling times and phenotypic features. This observation is supported by a comprehensive multi-omics analysis by Liu et al., which revealed marked heterogeneity among HeLa cell lines with significant differences in protein expression and growth dynamics (Liu et al., 2019). These findings underscore the intrinsic variability of widely used cancer cell lines and the need for careful interpretation of experimental results.

Given this variability, the impact of transitioning from FBS-supplemented media to CDM on HeLa cell doubling times may be more pronounced in other laboratories than observed in our study. In such cases, a stepwise or extended adaptation process – gradually weaning cells off FBS – may improve cellular performance in CDM. This strategy may also be beneficial for more demanding experimental applications. However, for routine purposes such as educational use or basic functional assays (e.g., luciferase activity, siRNA efficacy, or transfection efficiency), direct adaptation is typically sufficient, with cells generally performing reliably after a few passages in CDM. Nonetheless, cells undergoing adaptation may exhibit morphological changes and altered growth characteristics, including reduced proliferation rates compared to those maintained in FBS-supplemented medium. In such cases, adjustment of the passaging procedure based on the counted cell numbers can help maintain optimal cell viability and experimental consistency.

The CDM formulated for the laboratory course includes HEPES as a buffering agent. HEPES is a well-established component in cell culture media and offers clear advantages in settings with extended handling times outside the incubator, such as practical courses involving students with limited laboratory experience. However, students should be made aware that HEPES is a xenobiotic compound, and its inclusion may not always be desirable (Weber et al., 2022).

The course covers several of the most important techniques in modern cell and molecular biology, such as cell passaging, transfection, and RNAi-mediated silencing, all performed in FBS-free cell culture. RNAi is a modern and widely used method for sequence-specific gene silencing and has also emerged as a promising therapeutic strategy (Kurreck, 2009; Friedrich and Aigner, 2022; Liu et al., 2025). For the readout, dual-luciferase assays, qRT-PCR, and Western blotting experiments were carried out with HeLa cells cultured in CDM. Where needed, animal-derived reagents were replaced with viable alternatives. TrypLE, a recombinant substitute for porcine trypsin, performs comparably and is cost-competitive. Recombinant BSA and antibodies obtained by phage display also reduce animal reliance without compromising function. Students perform RNAi experiments targeting GP73 using siRNA transfection based on the study by Liu et al. (Liu et al., 2016). The Golgi-resident protein GP73 was selected as a target because it is a timely focus of cancer research, particularly with respect to its potential role in promoting tumor development (Feng et al., 2024). Ongoing studies are investigating its involvement in epithelial–mesenchymal transition (EMT), and it is increasingly regarded as a promising candidate for therapeutic intervention. The experiments demonstrated that RNAi and plasmid transfection are effective in cells cultured in CDM. Additionally, they introduce students to critical concepts in assay normalization, control use, and detection strategies.

The Western blot module includes monoclonal and recombinant antibodies, and an HRP-conjugated antibody generated by phage display, offering a practical entry point into the ethical and technical considerations of antibody use (Gray et al., 2020; Ayoubi et al., 2023; Kwon, 2024). The differences in production methods – polyclonal, monoclonal, recombinant, and phage display-derived (Guliy et al., 2023) – are discussed in relation to specificity, reproducibility, and animal welfare. In the experiment described here, a monoclonal primary antibody against GP73 was combined with a recombinant secondary antibody. The housekeeping protein actin is detected using an HRP-conjugated antibody generated through phage display technology. This fully animal-free antibody replaced the previously used combination of a monoclonal mouse anti-actin primary antibody and a polyclonal goat anti-mouse secondary antibody, a practice of particular ethical concern, as polyclonal antibodies must be newly generated in animals once depleted.

The course also encourages reflection on the broader prevalence of animal-derived reagents (Duarte et al., 2023) (Figure 1). For example, basement membrane extract (BME), such as Matrigel, derived from murine Engelbreth-Holm-Swarm (EHS) sarcoma, is widely used in 3D (Benton et al., 2014) culture and bioprinting despite ethical and scientific concerns. Alternatives exist but require active integration into lab practice (Aisenbrey and Murphy, 2020). Beyond BME, other animal-derived materials such as gelatin and collagen are also frequently used, particularly in bioprinting applications (Berg and Kurreck, 2021). In a recent systematic review of all published studies on liver tissue bioprinting, we found that none of the examined publications reported the use of fully animal-free conditions although bioprinted tissue models are widely considered an alternative to animal testing (Ali et al., 2024b). To demonstrate the feasibility of an animal-free approach, we recently developed and presented a completely xeno-free liver model, in which cells were maintained in a CDM and animal-derived components in the bioink were successfully replaced with animal-free alternatives (Ali et al., 2024a).

A major barrier to broader adoption of CDM is the continued reliance on FBS-based protocols, which are deeply entrenched in laboratory routines and often perpetuated by senior researchers. While the need to replace FBS and other animal-derived reagents – both to improve reproducibility and reduce reliance on animal products – is widely recognized, progress remains slow. As mentioned above, Max Planck famously observed that scientific paradigms often shift not through the immediate acceptance of new ideas, but as older generations of scientists retire or pass away – a sentiment often summarized as “science advances one funeral at a time.” To help catalyze this transition, we redesigned a graduate-level laboratory course to introduce students to animal-free, human-relevant cell culture systems. By providing a detailed standard operating procedure (SOP, Supplementary Material 2) and a comprehensive course script (Supplementary Material 1), we aim to facilitate adoption at other institutions and contribute to a broader shift toward ethical, reproducible, and modern biomedical research.

## Data Availability

The original contributions presented in the study are included in the article/Supplementary Material, further inquiries can be directed to the corresponding author.
