# Supplementary material for: Promoting ethical and reproducible cell culture: implementing animal-free alternatives to teaching in molecular and cell biology
Source: Front Toxicol. 2025 Oct 1;7:1670513. doi: 10.3389/ftox.2025.1670513 (PMC12521421; doi:10.3389/ftox.2025.1670513)
Supplement: Supplementary file 1 [file Supplementaryfile1.docx]

Supplementary Material

**Promoting Ethical and Reproducible Cell Culture: Implementing Animal-Free Alternatives to Teaching in Molecular and Cell Biology**

Alexandra Nessar^1^, Viola Röhrs^1^, Mathias Ziersch^1^, Ahmed S. M. Ali^1^, Julia Moradi^1^, Anke Kurreck^2,3^, Johanna Berg^1,4^, Jens Kurreck^1*^

^1^ Chair of Applied Biochemistry, Institute of Biotechnology, Technische Universität Berlin, Berlin, Germany

^2^ Chair of Bioprocess Engineering, Institute of Biotechnology, Technische Universität Berlin, Berlin, Germany

^3^ BioNukleo GmbH, Berlin, Germany

^4^ Research, Transfer and Startup Center, Anhalt University of Applied Science, Köthen, Germany

*** Correspondence:**Jens Kurreck
jens.kurreck@tu-berlin.de

**Content:**

Supplementary Figure S1. Osmolality of the media

Supplementary Material 1: Laboratory Course Script

Supplementary Material 2: SOP for HeLa cultivation in CDM

**Supplementary Material 3: Transcript of Surveys**

Supplementary Material 4: Cost of the Culture Media

**Supplementary Figure 1: Osmolality of the media**


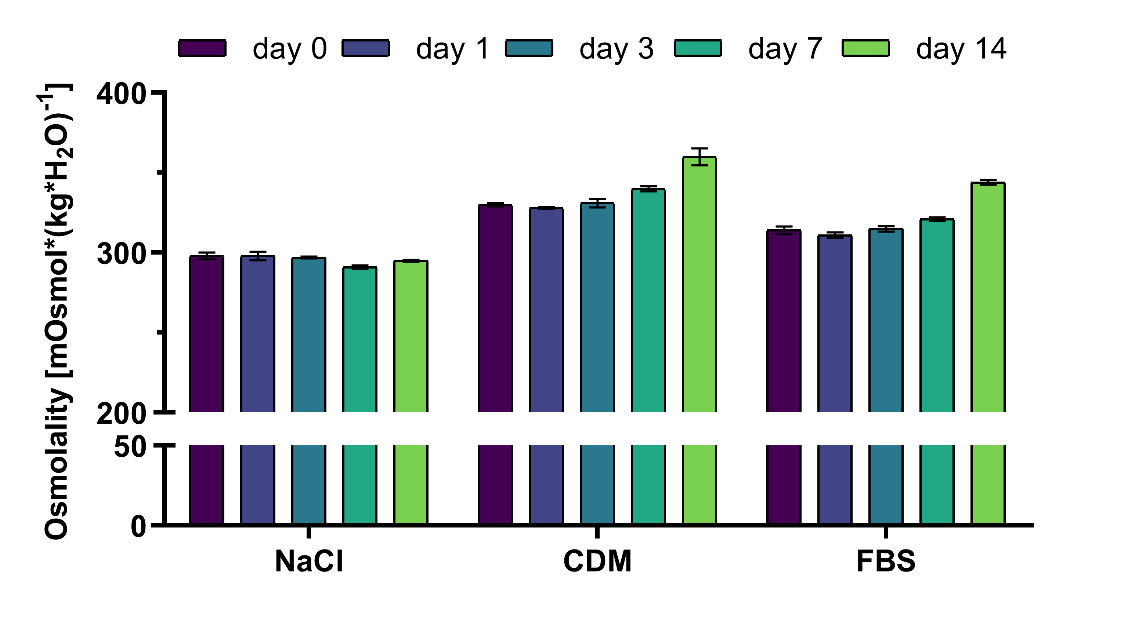


Supplementary Figure S1. Osmolality of FBS-free CDM and FBS-supplemented medium over a 14-day period. Osmolality measurements of chemically defined medium (CDM) and conventional medium supplemented with 10% FBS were monitored over 14 days. Both media showed a moderate increase in osmolarity over time. Physiological saline served as a stable reference control throughout the experiment. Data represent mean ± SD from three technical replicates.

**Supplementary Material 1: Laboratory Course Script**

**FBS-free Cell Culture Laboratory Course**

**Content**

[1 Group and experiment schedule 1](#_Toc198578841)

[2 Introduction to RNA interference 1](#_Toc198578842)

[3 Experiments 3](#_Toc198578843)

[3.1 Experiment 1: Transfection of a Cy3 modified siRNA 3](#_Toc198578844)

[A Seeding of HeLa cells 3](#_Toc198578845)

[B Transfection 4](#_Toc198578846)

[C Cell fixation and DAPI staining 7](#_Toc198578847)

[D Brain teasers 8](#_Toc198578848)

[E Notes Experiment 1 8](#_Toc198578849)

[3.2 Experiment 2: Dual Luciferase Assay (DLA) 9](#_Toc198578850)

[A Seeding of HeLa cells 10](#_Toc198578851)

[B Co-Transfection of siRNA and reporter plasmid (psiCheck™II-GP73) 10](#_Toc198578852)

[C Lysis of the cells and measurement of luciferase activity 12](#_Toc198578853)

[D Notes Experiment 2 12](#_Toc198578854)

3.3 [Experiment 3: Quantitative RT-PCR (qRT-PCR) 13](#_Toc198578855)

[A Carrying out qRT-PCR 13](#_Toc198578856)

[B Analysis 15](#_Toc198578857)

[C Additional in-depth tasks qPCR 15](#_Toc198578858)

[D Notes Experiment 3 17](#_Toc198578859)

[3.4 Experiment 4: Knockdown of GP73 in HeLa cells with siRNA 18](#_Toc198578860)

[A Seeding of HeLa cells 19](#_Toc198578861)

[B Knockdown of GP73 19](#_Toc198578862)

[C Preparation polyacrylamide gels 21](#_Toc198578863)

[D Cell lysis 22](#_Toc198578864)

[E Electrophoresis 23](#_Toc198578865)

[F Western blot 24](#_Toc198578866)

[G Blocking, antibody incubation and chemiluminescence detection 25](#_Toc198578867)

[H Notes Experiment 4 26](#_Toc198578868)

[4 Appendix 27](#_Toc198578869)

[4.1 References 27](#_Toc198578870)

[4.2 Protein Standard 27](#_Toc198578871)

[4.3 Dual Luciferase Reporter Assay Solutions - Hampf and Gossen, 2016 (2) 28](#_Toc198578872)

# Group and experiment schedule

Please read through the current section of the experiment before starting the corresponding work. It is your responsibility to discuss any questions you might have with your experiment supervisor and your group **before** starting the work. Come to an agreement within your group so that you are each responsible for carrying out your experiments independently and accurately. Concentrate on the correct execution of the experiment during the experiment and discuss it as comprehensively as possible with your fellow students. Work across groups to optimally link the individual sub-experiments. Provide the other groups with your results or give them to the experiment supervisors so that they can be shared internally. Please use the time when you are not working experimentally to organize yourself within the group to plan your own breaks and prepare presentations (Figure 1).

Figure 1: Overview of all experiments

# Introduction to RNA interference

RNA interference (RNAi) is a molecular mechanism that is essential for the regulation of gene expression in eukaryotic organisms. Discovered late in the 20th century, RNAi has become an important tool in molecular biology, enabling precise control of gene function. At its core, RNAi controls the silencing of certain genes through the degradation of complementary mRNA molecules. This complex regulatory pathway not only plays a central role in cellular homeostasis but also harbors enormous potential for therapeutic intervention. In this practical course, you will familiarize yourself with various properties and possible applications of RNAi strategies.

One way to prevent protein translation is to use antisense oligonucleotides. This antisense strategy usually involves the use of short oligodeoxynucleotides that are complementary to the mRNA of the target molecule. The resulting complex of DNA and RNA inhibits translation and is degraded by the cell's own RNAases so that the protein can no longer be synthesized. In the mid-1990s, it was shown that single-stranded DNA can also act as an enzyme and degrade RNA.

Another very efficient method for silencing genes is RNA interference, in which double-stranded RNA is used. Two primary pathways, namely the small interfering RNA (siRNA) pathway and the microRNA (miRNA) pathway, describe the complex mechanisms of RNAi. The siRNA pathway begins with the introduction of double-stranded RNA (dsRNA), which is either of exogenous origin or a by-product of endogenous processes. In contrast, the miRNA pathway starts from endogenously encoded transcripts that fold into hairpin structures and form precursor miRNAs (pre-miRNAs). Both siRNA and miRNA pathways converge in the RNA-induced silencing complex (RISC) and serve as central effectors for RNA silencing (Figure 2).


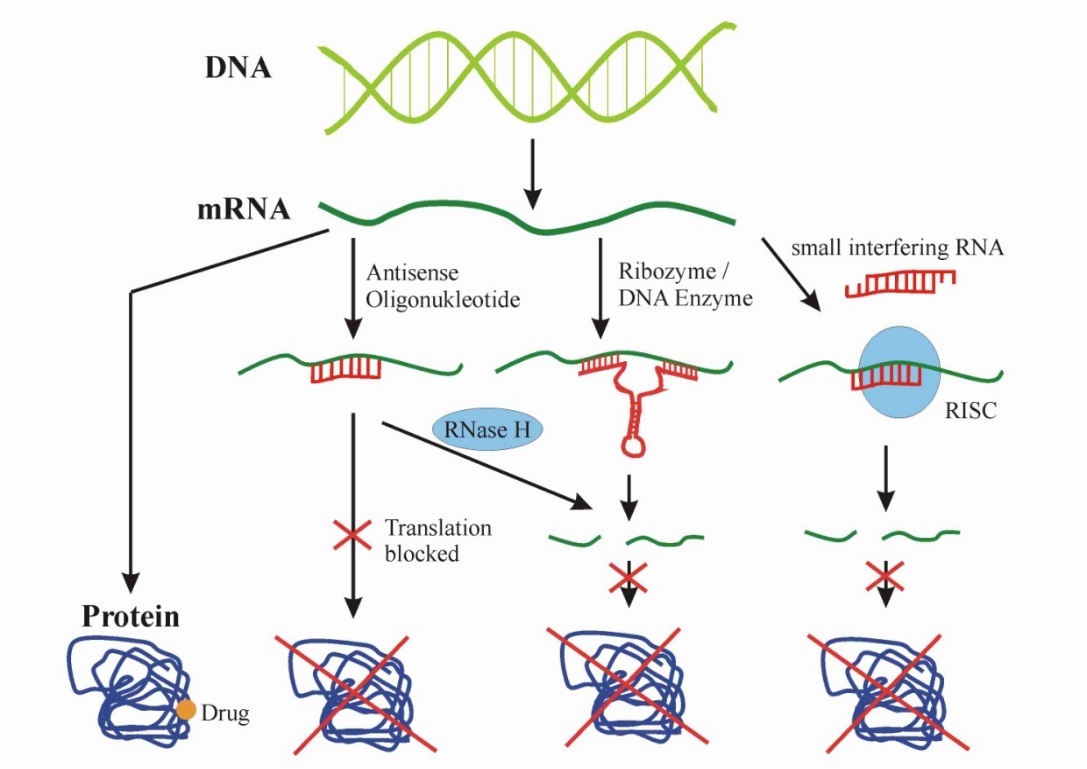


Figure 2: Function of antisense oligonucleotides, ribozymes/DNAzymes and siRNA [Kurreck, J. (2003) Eur. J. Biochem. 270,1628–1644]

In lipotransfection, liposomes encapsulate the genetic material and fuse with the cell membrane, facilitating the transfer of the genetic “cargo” into the cell. The liposomes protect the genetic material from degradation and help it to enter the target cells. Researchers often use lipotransfection to study gene function, manipulate cellular processes and develop potential therapeutic treatments for genetic disorders or diseases. In experiment 1, you will learn how to introduce siRNAs into eukaryotic cells and test the appropriate conditions (transfection reagents and siRNA concentration). In experiment 2, you will determine the efficiency of an siRNA using a dual-luciferase assay. The quantitative polymerase chain reaction (qPCR) will be employed in experiment 3 in order to efficiently determine RNA amounts. Here it must be considered that the quantification of RNA can only be carried out after previous reverse transcription (RT) of RNA to cDNA (qRT-PCR). Due to time constraints, the transfection, isolation of the RNA, DNA degradation and RT will be carried out by the technical assistant prior to the practical course. An actual knockdown experiment by an siRNA will then be carried out in experiment 4 and its success checked at the protein level via Western blot and immunostaining. The siRNAs and their corresponding sequences can be found in Table 1 and will be used in the following experiments.

Table 1: siRNA sequences used for target gene knockdown and functional studies.

| **siRNA** | **5‘ 🡒 3‘Sequence** | **Usage** |
| --- | --- | --- |
| siGP73 | Sense CAA GCU GUA CCA GGA CGA A [dTdT]  Antisense UUC GUC CUG GUA CAG CUU G [dTdT] | siRNA targeting GP73 - (1) |
| siCon | Sense UUC UCC GAA CGU GUC ACG U [dTdT]  Antisense ACG UGA CAC GUU CGG AGA A [dTdT] | Non-binding control siRNA  (negative control) |
| siCon-Cy3 | Sense UUC UCC GAA CGU GUC ACG U [dTdT]  Antisense ACG UGA CAC GUU CGG AGA A [dTdT] | Non-binding control siRNA with Cyanine3 (Cy3) modification |

# Experiments

The aim of experiments 1-4 is to apply basic techniques of transfection of genetic material into eukaryotic cells and the necessary detection methods such as fluorescence microscopy, luciferase assay and Western blot. In addition, you will learn about the relationship between the quality of your cell culture work and the quality of your experimental results. The HeLa cells in this practical course are cultivated under FCS-free conditions in chemically defined medium (CDM), which you will receive from our cell culture.

For cell culture experiments on well plates, it is essential to correctly classify and use the standard units for cell numbers. Transfection protocols typically specify the required number of cells per well for a given assay (e.g. 2×10⁵ cells per well). However, for general cell culture procedures, especially during dilutions, cell counts are expressed as cells per milliliter. Both units must be used correctly and documented accordingly. Each step should be clearly noted with the appropriate units. The instructors are available to provide further clarification, if needed

## Experiment 1: Transfection of a Cyanine3 (Cy3) modified siRNA

In cell biology, transfection refers to the introduction of a foreign DNA or RNA into a eukaryotic cell; various methods have been described for this purpose. The K4 Transfection System from Biontex is used to knock down the target protein GP73 in HeLa cells. It uses cationic lipids in an aqueous solution to form liposomes with negatively charged (anionic) genetic material to be transfected into the eukaryotic cells. It will be compared to TurboFect™ (polyethylene) from Thermofisher Scientific. Through electrostatic forces, they condense with the negatively charged phosphate groups on the DNA and thus form lipoplexes or polyplexes, which can be taken up by the cell through endocytosis. The various cationic polymers differ from each other in length, geometry, and functional groups.

Figure 3: Flow chart showing tasks for groups 1 & 2, as well as 3 & 4.

In this experiment, you will introduce a Cy3 coupled siRNA into HeLa cells. You then fix the cells, stain their nuclei with DAPI and prepare permanent preparations. To be able to make a statement about the transfection efficacy of the reagents, compare the results of your individual experiments with those of the other groups.

### Seeding of HeLa cells

Each group requires **6-8 wells with 1×10^5^** cells seeded each on a 24-well plate for its part of the experiment. To avoid confusion, each group will work with its own plate. The instructions for preparing the cells and seeding them are separately supplied.

**Important Information:** The cells from experiments 1 and 2 (Dual Luciferase Assay) are seeded at the same time. Make sure you know how many wells you need to prepare for **both** experiments.

To prepare permanent preparation after the transfection, the cells must be grown on round cover slips, which are coated with a layer of human placenta collagen (The Human Touch Biomaterials, Vienna , Austria) before seeding into the wells (Figure 4). All this work must be carried out under sterile conditions under a laminar air flow bench. Before seeding the cells, please discuss the practical implementation within your group.

Figure 4: Collagen coating cover slips and cell seeding for transfection

When cells are handled outside the incubator, they are exposed to non-physiological temperature and suboptimal gas conditions, which can compromise viability. As certain procedures in a teaching laboratory may take 45–90 minutes per group, cells are frequently outside their optimal environment. Therefore, whenever possible, cultures should remain in the incubator when not actively being manipulated. Before preparing the working solution for counting and seeding, the cells are resuspended by pipetting them up and down with a serological pipette to separate them.

### Transfection

The seeded HeLa cells are assessed microscopically for their confluence, which should be >90% before the transfection. Further evaluation criteria to be recorded are: The uniformity of distribution, the general condition of the cells and the color of the medium. According to the following pipetting scheme, test the transfection reagents under different conditions.

**Groups 1 and 2 test two different siRNA concentrations.**

Figure 5: siRNA Transfection with K4-Reagent

Figure 5 illustrates the procedure and reagent quantities required for a single transfection reaction. In this experiment the efficacy of two different siRNA concentrations will be evaluated. The siRNA transfections are performed in duplicate. When preparing the transfection solution with siRNA for two wells, prepare a 2.5-fold volume. This excess is intended to compensate for potential pipetting errors or inaccuracies, as outlined in Table 2. The master mix containing the K4 reagent is prepared beforehand (5.5-fold volume), gently mixed, and incubated for 5 minutes before being added to the siRNA. The complete transfection mix should then be incubated for 15–20 minutes. Carefully pipette 50 µl of the transfection solution (for mock control use OptiMEM) dropwise into each well. Mark the well plate lid of those wells which were transfected with each transfection mix, as shown in Figure 6. Incubate the cells at 37°C for 24 hours until cell fixation.

Table 2: Transfection of siRNA in different concentrations.

|  | **per Well** | | | **2.5 Wells** | **2.5 Wells** |
| --- | --- | --- | --- | --- | --- |
| **Components** | **Starting**  **concentration** | **Final concentration** | ***in 550 µl*** | **A**  **siCon-Cy3: 50 nM** | **B**  **siCon-Cy3: 10 nM** |
| siCon-Cy3 | 10 µM | 50 nM | µl | **µl** |  |
| siCon-Cy3 | 1 µM | 10 nM | µl |  | **µl** |
| *add Opti-MEM for final volume* | | | | **µl** | **µl** |
|  |  |  |  | **62.5 µl** | **62.5 µl** |
|  |  |  |  | + | + |
| **Tube E** = K4 reagent mix for 5.5 wells = **µl** Opti-MEM +  **µl** K4 | | | | **62.5 µl** | **62.5 µl** |

Figure 6: Pipetting scheme (groups 1 and 2)

**Groups 3 and 4 test two different transfection reagents with the same siRNA concentration.**

Figure 7: siRNA Transfection with K4-Reagent and TurboFect

Figure 7 illustrates the procedure and reagent quantities required for a single transfection reaction. In this experiment two different transfection reagents will be compared. The siRNA transfections are performed in duplicate. Additionally, prepare extra volume (2.5 wells) to account for potential pipetting errors or inaccuracies, as shown in Table 3. The mix containing the K4 reagent or TurboFect reagent is prepared beforehand (2.5 wells) and incubated for 5 minutes before being added to the siRNA. Carefully pipette 50 µl of the transfection solution (for mock control use OptiMEM) dropwise into each well. Note on the well plate lid which wells were transfected with each transfection mix, as shown in Figure 8. Incubate the cells at 37°C for 24 hours until cell fixation.

Table 3: Transfection of siRNA with different transfection reagents.

|  | **1 Well** | | | **2.5 Wells** | **2.5 Wells** |
| --- | --- | --- | --- | --- | --- |
| **Components** | **Starting**  **concentration** | **Final concentration** | ***in 550 µl*** | **A**  **K4 Reagent** | **B**  **TurboFect** |
| siCon-Cy3 | 10 µM | 50 nM | µl | **µl** | **µl** |
| *add Opti-MEM for final volume* | | | | **µl** | **µl** |
|  |  |  |  | **62.5 µl** | **62.5 µl** |
|  |  |  |  | + | + |
| **Tube E** = K4 reagent mix for 2.5 wells = **µl** Opti-MEM +  **µl** K4 | | | | **62.5 µl** |  |
| **Tube F** = TurboFect mix for 2.5 wells = **µl** Opti-MEM +  **µl** K4 | | | | µl | **62.5 µl** |

Figure 8: Pipetting scheme (groups 3 and 4)

### Cell fixation and DAPI staining

To evaluate the efficacy of the transfection, the transfected cells must be fixed on glass slides with mounting medium (Figure 9). To fix the cells to the cover slips, the medium is removed completely and the cells are carefully washed with PBS. Avoid harsh aspiration or rinsing. Then 200 μl of 4% formaldehyde is added to each well. The plate is incubated for 15 minutes at room temperature. To prevent unnecessary evaporation, cover the plate with a lid. The fixative solution is then removed, and the wells are rinsed twice with PBS. This step must not damage or detach the cell monolayer. Using tweezers, carefully remove the coverslips from the wells and dry them by placing them on a paper towel with the cells facing upwards for approximately 10 minutes.

To preserve the preparation, place a small drop of mounting medium (ROTI®Mount Flour Care DAPI) on a microscope slide and carefully position the coverslip on top with the cells facing downwards, ensuring no air bubbles are trapped. The resin polymerizes in the absence of air and contains DAPI (4′,6-diamidino-2-phenylindole).

DAPI dye is used in fluorescence microscopy to stain cell nuclei. The compound preferentially binds to the minor groove of DNA. When bound to double-strand DNA, its absorption maximum is at a wavelength of 358 nm, and its emission maximum is at 461 nm. In addition to DAPI staining, Cy3-labeled siRNA is also evaluated. Cy3 is a fluorescent dye with an absorption maximum at 550 nm and an emission maximum at 570 nm, allowing visualization of siRNA localization within the cells.

Figure 9: Preparation of fixed cells and staining them with DAPI

### Brain teasers

- What effect would it have if your sterile technique fails while seeding the cells and you accidentally introduce fungi or bacteria into the well? How can you recognize such a failure?
- What factors play an important role in the cultivation of cells with respect to obtaining reproducible results?
- Why do you use medium without antibiotics in this experiment?
- Which parameters determine the concentration of the transfection reagents in the experiment?In this experiment you use differing amounts of transfection reagents, although you want to compare both substances in terms of their efficacy. How can you explain this?
- After mixing the transfection reagent and siRNA, you must wait 20 minutes before applying them to the cells. Why?
- Why is a homogeneous cell monolayer crucial for the evaluation of your experimental results?
- You should record any deviations from the protocol. Explain why this is important and in which case it can be decisive for your work.
- Describe the difference between fluorescence and luminescence.

### Notes Experiment 1

## Experiment 2: Dual Luciferase Assay (DLA)

In this experiment, the efficiency of an siRNA will be tested. Whether the respective siRNA handed out by the instructor is effective, i.e. whether it binds to the selected target sequence, will be tested in a dual luciferase assay as part of this experiment. This requires preliminary work in molecular biology and cell biology: To carry out the dual luciferase test, a vector (psiCheck™2) is first cloned. Once cloning is complete, this vector contains the siRNA target sequence, a DNA sequence for Renilla and Firefly luciferase and one for ampicillin resistance. After successful cloning, the target sequence is located within the 3'UTR sequence of Renilla luciferase, between the functional mRNA and its polyadenylation site. Luciferases use specific substrates to trigger bioluminescence. While Renilla luciferase originates from a specific species of coral, Firefly luciferase is produced by the firefly beetle. This vector is transfected together with the designed siRNA into eukaryotic cells by using a transfection reagent that enables the uptake of the vector and siRNA together into the cells. After 24h, the actual dual-luciferase assay is performed. The expression and activity of both Renilla and Firefly can be detected indirectly by a luminometer (Tristar‑5, Berthold Technology, Bad Wildbad, Germany) as light signals (Figure 10). For internal normalization the ratio of Renilla to Firefly is formed. Higher concentrations of siRNA should result in a decreased signal.

Figure 10: Bioluminescence reactions catalyzed by Firefly and Renilla luciferases. Bettle luciferin is substrate for the firefly luciferase and coelenterazine is substrate for renilla luciferase. Both reactions emit photons (light), which can be detected with a luminometer (Technical Manual, Nano-Glo® Dual-Luciferase® Reporter Assay System, Promega, Madison, WI, USA).

**Preliminary molecular biological work**

The dual-luciferase assay requires a plasmid that contains the target DNA and the reporter genes Renilla (hRluc) and Firefly (hluc +) in addition to the usual plasmid components. The plasmid for this experiment was created by ligating the target sequence with the commercially available plasmid psiCheck™-2 from Promega, whose plasmid map is shown below in Figure 11. Restriction digestion of the ring-shaped vector was carried out using the two restriction enzymes NotI and XhoI. This enabled the insertion of the target sequence into the vector. The successfully created plasmid was propagated by transformation into *E. coli*. Successful testing of the siRNA using the dual-luciferase assay requires isolation of the plasmids from the *E. coli* culture. Since only a small amount of plasmid is required, a so-called midi-prep is suitable, for which a kit from the company Macherey-Nagel (NucleoSpin® Plasmid EasyPure, Düren, Germany) was used. Due to time constraints, the instructor will have prepared a suitable amount of the vector in advance.

Figure 11: psiCHECK™2. The vector shown is used as the starting plasmid for the dual luciferase assay. Restriction digestion with the enzymes NotI and XhoI enables cloning of the target sequence as an insert into the plasmid. This is then transfected together with an siRNA (psiCHECK™2, Promega).

### Seeding of HeLa cells

Each group needs a minimum of 12 wells with 1**×**10^5^ cells each on a 24-well plate (Techno Plastic Products AG, Trasadingen, Switzerland) for their part of the experiment. To avoid mistakes, each group will work with its own plate. The instructions for preparing the cells and seeding them can be found separately. **The cells for experiments 1 and 2 must be seeded at the same time.**

### Co-Transfection of siRNA and reporter plasmid (psiCheck™2-GP73)

The transfection takes place into the HeLa cells seeded the previous day. The confluence should be approx. >90% on the day of transfection. The plasmid containing the siRNA target sequence and the respective siRNA serve as starting material for the transfection.

Figure 12: DLA transfection with the K4-Reagent

The procedure and reagent quantities required for a single transfection reaction is illustrated in Figure 12. All siRNA concentrations are tested in triplicate. Additionally, prepare an extra volume (3.5 wells) to account for potential pipetting errors or inaccuracies, as shown in Table 4. The master mix containing the K4 reagent is prepared beforehand (15 wells), gently mixed, and incubated for max. 5 minutes before being added to the siRNA and vector preparation. The complete transfection mix should then be incubated for 15–20 minutes. Carefully pipette 50 µl of the transfection solution dropwise into each well. Note on the well plate lid which wells were transfected with each transfection mix, as shown in Figure 13. Incubate the cells at 37°C for 24 hours, when cell lysis is carried out.

Table 4: Co-transfection of psiCHECK™-2-GP73 vector and siRNA.

|  | **per Well** | | | **3.5 Wells** | **3.5 Wells** | **3.5 Wells** | **3.5 Wells** |
| --- | --- | --- | --- | --- | --- | --- | --- |
| **Components** | **Starting**  **concentration** | **Final concentration** | ***in 550 µl*** | **Tube A**  **10 nM** | **Tube B**  **1 nM** | **Tube C**  **0.1 nM** | **Tube D**  **siCon** |
| siGP73 | 10 µM | 10 nM | **µl** | **µl** |  |  |  |
| siGP73 | 1 µM | 1 nM | **µl** |  | **µl** |  |  |
| siGP73 | 0.1 µM | 0.1 nM | **µl** |  |  | **µl** |  |
| siCon | 10 µM | 10 nM | **µl** |  |  |  | **µl** |
| psiCHECK™-2-GP73 | 100 ng/µl | 250 ng/well | **µl** | **µl** | **µl** | **µl** | **µl** |
| *add Opti-MEM for final volume* | | | | **µl** | **µl** | **µl** | **µl** |
|  |  |  |  | **87.5 µl** | **87.5 µl** | **87.5 µl** | **87.5 µl** |
|  |  |  |  | + | + | + | + |
| **Tube E** = K4 reagent mix for 15 wells = **µl** Opti-MEM +  **µl** K4 | | | | **87.5 µl** | **87.5 µl** | **87.5 µl** | **87.5 µl** |

Figure 13: Experiment 2 suggested pipetting scheme

### Lysis of the cells and measurement of luciferase activity

The cells are checked microscopically before lysis. A confluent cell monolayer should be present. The medium should not be cloudy and should contain only a few floating cells. The medium is removed, and the cells are washed carefully once with PBS. Add 150 μl of 1X passive lysis buffer (Promega) per well to the cells and incubate the plate for 20 minutes at room temperature with shaking. The cell lysates are carefully transferred to appropriately labeled reaction vessels. If the cell monolayer has not completely detached, the lysate can be pipetted up and down several times to detach them.

The cell suspension is then mixed well, centrifuged and 10 μl of sample is pipetted into a white 96-well plate. The Renilla and Firefly activity is measured automatically in a luminometer (TriStar-5, Berthold Technologies GmbH).

The substrate solutions have already been prepared according to the protocol by Hampf and Gossen (2) and loaded into the luminometer by your instructor. Their composition can be found in section 4.3 of the appendix. For each measurement step, 50 μl of substrate solutions are automatically dispensed. Signal integration was performed for 10 seconds per measurement, with a 2-second delay after each injection.

You will receive a data file of the measured values from which the ratio of firefly signal and renilla signal can be calculated (internal normalization). Investigate the silencing of the siRNA compared to the control by normalizing the measured values.

### Notes Experiment 2

## 3.3 Experiment 3: Quantitative RT-PCR (qRT-PCR)

The polymerase chain reaction (PCR) is a method for the rapid and uncomplicated amplification of DNA sequences.The method makes use of the fact that the amplification efficiencies in the exponential phase of PCR are approximately constant. This makes it necessary to determine when each individual reaction leaves the exponential phase of the PCR reaction. The double-stranded DNA can be detected using the intercalating dye SYBR Green I.

By measuring the fluorescence light intensity, which is proportional to the amount of product, this can be done comparatively easily and without interference. This measurement is carried out in real time during the PCR and is therefore also referred to as real-time PCR. At the beginning of the PCR, in the exponential phase, the double-stranded PCR product theoretically doubles with each cycle. While the reactions are never 100% efficient, in practice the amount of product comes close to doubling. Thus, the fluorescence signal increases proportionally to the amount of PCR product with each cycle.

Figure 14: DNA SYBR Green I. A ) Excitation and emission spectra of SYBR Green I. B) Schematic illustration how SYBR Green I interacts with double stranded DNA while undergoing the polymerase chain reaction.

### Carrying out qRT-PCR

You will be supplied with cDNA samples prepared prior to the course. HeLa cells were transfected with siRNA against GP73 (10 nM and 50 nM) and siCon (random control, 50 nM). After transfection, all samples were incubated at 37°C for 48 hours. The RNA was isolated, followed by DNA digestion and reverse transcription into complementary DNA (cDNA). The expression of GP73 can now be quantified using qPCR.

Table 5: Primers for quantitative PCR

| **Primer** | **5‘ 🡒 3‘sequence** | **Melting temperature**  **(T_m_) in °C** |
| --- | --- | --- |
| GP73_fwd | CAG CGC TGA TTT TGA GAT GAC | 64.5 |
| GP73_rev | ATG ATC CGT GTC TGG AGG TG | 66.3 |
| 18S_fwd | CGC GGT TCT ATT TTG TTG GT | 64.2 |
| 18S_rev | AGT CGG CAT CGT TTA TGG TC | 65.1 |

The reaction volume of the qPCR should be 20 μl. All samples (siCon 50 nM, siGFP73 with 10 nM and 50 nM) should be measured twice independently. Perform the PCR for the gene of interest (GOI) and a housekeeping gene (18S) as the internal control. Prepare the two master mixes (8 reactions each) with all components except the template cDNA. This includes a no template control (NTC) in which you add ddH_2_O (Milli-Q) instead of cDNA. Use the following Table 6 to calculate the necessary volumes.

Table 6: Preparation of PCR

| **Components** | **Start**  **concentration** | **Final**  **concentration** | **DF** | **Volume**  **1 reaction** | **Volume**  **master mix** |
| --- | --- | --- | --- | --- | --- |
| SsoFast EvaGreen Mastermix (Bio-Rad) | 2X | 1X | 2 | µl | µl |
| Template (cDNA) | ----- | ----- | ----- | 2 µl |  |
| forward Primer | 10 µM | 0.4 µM | 25 | µl | µl |
| reverse Primer | 10 µM | 0.4 µM | 25 | µl | µl |
| H_2_0 | ----- | ----- | ----- | µl | µl |
|  |  |  | Total volume | 20 µl | µl |

A pipetting template for the qPCR well plate will be provided by your instructor to ensure consistency across all groups. This will simplify data analysis later. Mark the desired wells with a pen and add 18 μl of the master mixes to each well on the plate. Then, pipette 2 μl of cDNA sample or water (NTC) into each corresponding well. Finally, place the well plate in the qPCR cycler (CFX96 Real-Time System, Bio-Rad) and use the amplification program in Table 7 below:

Table 7: Thermal cycling conditions for PCR

| **Cycle stage** | **Temperature** | **Time** | **Cycles** |
| --- | --- | --- | --- |
| Initial Denaturation | 95°C | 30 s | 1x |
| Denaturation | 95°C | 5 s | 40x |
| Annealing / Elongation | 60°C | 5 s |  |
| Plate read | *fluorescence intensity* | |  |
| Denaturation | 95°C | 5 s | 1x |
| Melting Curve Analysis | 65°C -> 95°C. increase: 0.5°C/s +plate read | | 1x |
| Storage | 4°C | ------ | ------ |

### Analysis

To analyze the qPCR data, a threshold value is set within the exponential increase of the signal curve. The C_T_ value (threshold cycle) can be read at the intersection of the signal curve with the threshold value. The higher the copy number of the cDNA template available for amplification at the beginning, the earlier there will be a significant increase in the fluorescence signal and the corresponding C_T_ value will be lower, reflecting a signal in an earlier cycle (Figure 15A).

For a quantitative analysis of an unknown sample, a linear regression is usually used, graphically represented as C_T_ values against the previously selected logarithmic starting quantity/copy number. The copy number of an unknown sample can be determined by rearranging the linear regression formula for y (Figure 15B).

In the case of optimal multiplication, the copy number doubles in each cycle, resulting in the following for the standard curve: PCR efficiency = 100%, correlation coefficient = 1, slope = -3.32, The efficiency is calculated by the following equation:

$$efficiency ={10}^{\frac{-1}{\mathrm{slope}}}-1$$


Figure 15: Data analysis after complete qPCR run. A) Fluorescence signal of the amplification of a serial dilution of a cDNA template. B) Linear regression of the C_T_ value against logarithmic initial concentration or quantity.

### Additional in-depth tasks for qPCR

Calculate the difference between two C_T_ values (using the PCR equation below) for an experiment in which the amount of DNA differs by a factor of 10.

PCR equation: y = x * V^n^

(x = Initial quantity, V = Multiplication factor per cycle, n = Number of cycles)

**Relative quantification**

In relative qRT-PCR, the expression of a gene-of-interest (GOI) is compared between two conditions after normalization against a reference gene. The so-called housekeeping genes such as β-actin, glyceraldehyde-3-phosphate dehydrogenase (GAPDH), ribosomal RNA such as 18S rRNA or phosphoribosyl-transferase serve as reference genes. An important prerequisite here is that the expression of these genes does not change under the experimental conditions.

2^-ΔΔCT^ method after Livak (3)

The ratio in the GOI expression is usually calculated using the ΔΔCT method:

| Normalization of individual C_T_ values:  Condition A e.g. normal cells (control)  Condition B: e.g. tumor cells (test) | ΔC_T(A)_ = 𝐶_𝑇 (𝐺𝑂𝐼 𝐴)_ - C_𝑇 (reference 𝐴)_  ΔC_T(B)_ = 𝐶_𝑇 (𝐺𝑂𝐼 𝐵)_ - C_𝑇 (reference B)_ |
| --- | --- |
| Normalization of the C_T_ values of  Condition B compared to A: | ΔΔC_T_=Δ𝐶_𝑇(𝐵)_ - Δ𝐶_𝑇(𝐴)_ |
| Calculation of the expression ratio | 2^−(ΔΔCT)^ |

**Example task (Table 8):**

Calculate the difference in the expression of TNFα using the housekeeping gene GAPDH, listed in the following table:

Table 8: Exemplary CT values after performing a qPCR to calculate the expression of TNFα

|  | **Control 1** | **Control 2** | **Control 3** | **Sample 1** | **Sample 2** | **Sample 3** |
| --- | --- | --- | --- | --- | --- | --- |
| **GAPDH** | 17.2 | 18.0 | 19.0 | 17.0 | 18.0 | 17.5 |
| **TNFα** | 31.0 | 32.3 | 33.4 | 26.0 | 27.2 | 26.8 |

**Calculations Experiment 5 (Table 9):**

- Analyze the relative expression of your GOI with the data from your qPCR run.
- Analyze the melting curve and discuss your results.

Table 9: Table for entering the data of your own qPCR run

|  | **siCon** | **siCon** | **10 nM siGP73** | **10 nM siGP73** | **50 nM siGP73** | **50 nM siGP73** |
| --- | --- | --- | --- | --- | --- | --- |
| **18S** |  |  |  |  |  |  |
| **GP73** |  |  |  |  |  |  |

### Notes Experiment 3

## 3.4 Experiment 4: Knockdown of GP73 in HeLa cells with siRNA

Golgi phosphoprotein 2 (GP73 or GOLPH2) is a transmembrane glycoprotein that occurs in the Golgi apparatus and has been shown to be involved in chronic inflammatory processes and carcinogenesis. Studies have already shown that GP73 can be used as a maker for hepatocellular carcinomas (HCC). In particular, there is a correlation between increased serum levels and liver fibrosis or cirrhosis. The expression of GP73 is low and strictly regulated in non-malignant tissues (4).

Knockdown of GP73 does not appear to directly suppress proliferation, but instead has an effect on cell motility. Results suggest that GP73 knockdown by siRNA may be a novel, low-toxicity therapy to inhibit tumor proliferation and metastasis (1).

Figure 16: Experiment 4 workflow

The aim of the experiment is to knock down the target protein GP73 using an siRNA and to evaluate the result qualitatively and, if possible, quantitatively. In this experiment, the siRNA is transfected into HeLa cells, a cervical cancer cell line, initially taken in 1951 from Henritta Lacks, a 31-year-old African American woman.

This experiment is carried out over the entire duration of the practical course and is therefore the most time-consuming. The individual experimental steps are shown in the flow chart in Figure 16. Each group carries out the experiment as a single experiment and compares its results with those of the other groups. You will therefore receive results from multiple biological experiments, which you should discuss accordingly in your evaluation.

### Seeding of HeLa cells

Each group performs the transfection on its own 12-well plate with a single siRNA. Including the controls, this will require a minimum of six wells, each seeded with 2**×**10^5^ cells in 1 ml medium. If possible, more wells will be prepared to allow consistent wells to be chosen to improve the results.

Every group will prepare their own dilutions based on their own cell count. Cell harvesting, cell counting, dilutions and seeding will be performed by each group independently. Transfection will be done 24h after seeding in the previously selected wells.

### Knockdown of GP73

| **Duration** | **ca. 2 hours** |
| --- | --- |
| Materials and chemicals | siGP73 10 μM  siCon 10 μM  K4 Transfection (Biontex, Munich, Germany)  OptiMEM |

The cells seeded the previous day will be assessed microscopically for confluence, general condition and uniformity of distribution before transfection and the results are recorded.

Figure 17 illustrates the procedure and reagent quantities required for a single transfection reaction. First, the necessary pre-dilutions of siRNA are prepared in 60 μl final volume each. The final concentrations used in the test are 10 nM, 50 nM and 80 nM. The master mix containing the K4 reagent is prepared beforehand (5.5X wells), gently mixed, and incubated for max. 5 minutes before being added to the siRNA in Table 10. The complete transfection mix should then be incubated for 15–20 minutes. Carefully pipette 120 µl of the transfection solution into each well by dripping. Mark on the well plate lid which wells were transfected with each transfection mix. as shown in Figure 18. Incubate the cells at 37°C for 48 hours until you are ready for the cell lysis step.

Figure 17: Western Blot transfection with the K4-Reagent

Table 10: Transfection of siRNA. All transfection mixtures are prepared for one well only.

|  | **1 Well** | | | **1 Well** | **1 Well** | **1 Well** | **1 Well** | **1 Well** |
| --- | --- | --- | --- | --- | --- | --- | --- | --- |
| **Components** | **Start**  **concentration** | **Final concentration** | ***in 1120 µl*** | **Tube A**  80 nM | **Tube B**  50 nM | **Tube C**  10 nM | **Tube D**  siCon | **Tube E**  only K4 |
| siGP73 | 10 µM | 80 nM | **µl** | **µl** |  |  |  |  |
| siGP73 | 10 µM | 50 nM | **µl** |  | **µl** |  |  |  |
| siGP73 | 10 µM | 10 nM | **µl** |  |  | **µl** |  |  |
| siCon | 10 µM | 80 nM | **µl** |  |  |  | **µl** |  |
| *add Opti-MEM for final volume* | | | | **µl** | **µl** | **µl** | **µl** | **µl** |
|  |  |  |  | **60 µl** | **60 µl** | **60 µl** | **60 µl** | **60 µl** |
|  |  |  |  | + | + | + | + | + |
| **Tube F:** K4 mix for 5.5 wells = **µl** Opti-MEM +  **µl** K4 | | | | **60 µl** | **60 µl** | **60 µl** | **60 µl** | **60 µl** |

Figure 18: Example of a pipetting scheme for experiment 4.

### Preparation of polyacrylamide gels

| **Time** | **ca. 2 hours** |
| --- | --- |
| Materials and chemicals | 30% acrylamide/bisacrylamid (29:1) solution  Resolving gel buffer 1.5 M Tris/HCI, pH 8.8  Stacking gel buffer 0.5 M Tris/HCI, pH 6.8  SDS-solution 10% in water  Ammonium persulfate (APS) 10% in water  Tetramethylethylendiamine (TEMED) |

Proteins from the cell lysate are separated using SDS-PAGE, with the acrylamide content adjusted based on protein size. The target protein (GP73) is 73 kDa. Polymerization is triggered with APS and TEMED, and the mixture stays liquid for about three minutes. Mix thoroughly while avoiding bubbles and foaming. Use designated pipettes labeled for acrylamide. Prepare only 10 ml for two gels at a time. Work quickly and carefully, ensuring the correct volume is reached before polymerization ensues. Plan what you are going to do carefully in advance and this step will go smoothly. You will have sufficient time to pour the gel, as long as you do not become distracted and pause. **Before starting the polymerization, please make sure the volume pipetted together up to this point is correct (Figure 19).**

Figure 19: Flow chart illustrating the production of SDS-PAGE gel preparation.

### Cell lysis

| **Time** | **approx. 60 minutes** |
| --- | --- |
| Materials and chemicals | RIPA Lysis-Buffer |

The HeLa cells are assessed microscopically before lysis and their condition is recorded. Pay particular attention to whether the appearance of the cells is consistent in the respective wells.

Carefully aspirate the medium and wash the cells with cold PBS. Add 100 µl of RIPA lysis buffer and incubate the samples on ice for about 5 minutes, swirling the plate occasionally. Gently resuspend the cell lysate. Take care not to introduce bubbles or loose material into the well. Freeze the cells in liquid nitrogen for 1-2 minutes and store your samples at -20°C until needed. Freezing and thawing further disrupts the cell walls. Centrifuge your sample at maximum g for 10 minutes to pellet all the unnecessary cell components. Store your sample on ice until it is used in the PAGE.

Viscous liquids are difficult to process. You need at least 10 μl to be able to carry out a PAGE run. A certain quantity and concentration of protein per gel pocket is necessary for the quality of separation quality.

The less viscous they are and the fewer air bubbles they contain, the easier they will be to apply. Viscosity can be reduced by cutting the end off of a pipette tip and pipetting the samples up and down several times or using a sonification probe (or bath). Your instructor will discuss these steps with you in detail and show you how to transfer the lysate as quickly and loss-free as possible.

Figure 20: Cell lysis for Western blot

### Electrophoresis

| **Time** | **approx. 3 hours** |
| --- | --- |
| Material  and methods | Loading buffer with 50 mM DTT  Electrophoresis running buffer 1X, pH 8.3, 0.025 M Tris, 0.192 M Glycine, 0.1 % SDS  Cozy™ Prestained Protein Ladder  Electrophoresis System (Biorad Mini-Protean, 1 mm) |

In SDS polyacrylamide gel electrophoresis, all proteins are denatured by the incorporation of negatively charged SDS (Sodium Dodecyl Sulphate) and given an identical charge density. This enables charge-independent separation of the proteins according to their size in a polyacrylamide network. A discontinuous gel is used, which consists of a stacking gel above the main separating gel, which leads to a better separation of the proteins. There are different buffer systems that have specific advantages depending on the separation problem. Most commonly used is the Laemmli system with Tris-glycine buffers. The cross-linking degree of the SDS gel is crucial for the separation performance in a certain molecular mass range.

The electrophoresis device used is a "Mini Protean 3 apparatus" from BioRad. The volume of running buffer must be adapted according to the electrophoresis system. The run is finished as soon as the dye has reached the bottom. Prepare the Western blot during the electrophoresis run.

Attention: The device reacts sensitively to residual currents, so work dry and carefully and check regularly whether it is still running

Figure 21: Operation of gel electrophoresis

### Western blot

| **Time** | **approx. 2-3 hours** |
| --- | --- |
| Materials and chemicals | Semi-Dry-Blot Buffer (transfer buffer) 47.9 mM Tris/HCl, 38.9 mM Glycine, 0.038 % SDS, 20 % v/v Methanol  TBS buffer (Tris-buffered-saline) 10X 200 mM Tris/HCl; 1.5 mM NaCl; pH 7.5  TBS-T 1X (1X TBS with 0.1 % Tween 20)  Blocking solution soy milk (low fat)  Primary antibodies mouse anti-GP73 (e.g. sc-393372, Santa Cruz Biotechnology)  Secondary antibodies Anti IgG 2a BP-HRP (sc-542732, Santa Cruz Biotechnology)  PVDF-Membrane (Roche Diagnostics)  ECL Western Blotting Substrate -Kit (Pierce, Thermo Fisher Scientific)  Methanol (blotting grade)  Blotting paper |

Figure 22: Semi-dry transfer preparation

The Western blot (immunoblot) is a method in which electrophoretically separated proteins are transferred from a separating gel to a suitable carrier (e.g. a membrane made of PVDF, nylon or nitrocellulose) by capillary action or electrophoresis. Electrophoretic transfer is performed using a semi-dry blot system.

Flip the PVDF membrane over once to ensure its complete activation with methanol. The methanol can be reused several times. The quality of the blot critically depends on the conditioning of the membrane with methanol. The entire surface of the membrane should be covered with it for about 30 seconds.

The blot is set up as shown in Figure 22. Avoid introducing air bubbles and make sure that all parts of the stack are wet evenly. The anode (bottom surface) must be wet so that the current can flow through the stack. Carefully place the lid on straight, avoid sideways or twisting movements and tighten the nuts of the apparatus evenly.

CAUTION:

Methanol is toxic and causes irreversible damage to organs. You use it in its pure state to prepare the membrane and as part of a blot buffer during electrophoresis. Both work steps must be carried out under a fume hood. Wear lab coats and gloves. Collect all methanol-containing waste in the appropriately labeled waste bottle. Allow methanol-infused wipes to evaporate under a running fume hood.

### Blocking, antibody incubation and chemiluminescence detection

In Western blotting, antibodies are used to specifically detect target proteins within a complex mixture. A primary antibody binds directly to the protein of interest, ensuring specificity. Since proteins are often in low abundance, a secondary antibody is used to amplify the signal. This secondary antibody is designed to recognize and bind to the primary antibody and is typically conjugated to horseradish peroxidase (HRP), which plays a crucial role in protein detection by catalyzing a chemiluminescent reaction when exposed to its substrate, such as luminol. This reaction produces light, which is captured using imaging systems or X-ray film, allowing the visualization of the protein bands. The intensity of the signal corresponds to the amount of target protein, making it possible to analyze protein expression levels. By using this antibody-based approach, Western blotting achieves high specificity and sensitivity in protein detection (Figure 23).

Figure 23: Membrane antibody binding steps with

subsequent chemiluminescence detection

### Notes Experiment 4

# Appendix

## References

1. Liu,Y., Zhang,X., Sun,T., Jiang,J., Li,Y., Chen,M., Wei,Z., Jiang,W. and Zhou,L. (2016) Knockdown of Golgi phosphoprotein 2 inhibits hepatocellular carcinoma cell proliferation and motility. *Oncotarget*, **7**, 21404–21415.

2. Hampf,M. and Gossen,M. (2006) A protocol for combined *Photinus* and *Renilla* luciferase quantification compatible with protein assays. *Analytical Biochemistry*, **356**, 94–99.

3. Livak,K.J. and Schmittgen,T.D. (2001) Analysis of Relative Gene Expression Data Using Real-Time Quantitative PCR and the 2−ΔΔCT Method. *Methods*, **25**, 402–408.

4. Liewen,H., Markuly,N., Läubli,H., Liu,Y., Matter,M.S., Liewen,N., Renner,C., Zippelius,A. and Stenner,F. (2019) Therapeutic Targeting of Golgi Phosphoprotein 2 (GOLPH2) with Armed Antibodies: A Preclinical Study of Anti-GOLPH2 Antibody Drug Conjugates in Lung and Colorectal Cancer Models of Patient Derived Xenografts (PDX). *Targeted oncology*, **14**, 577–590.

## Protein Standard


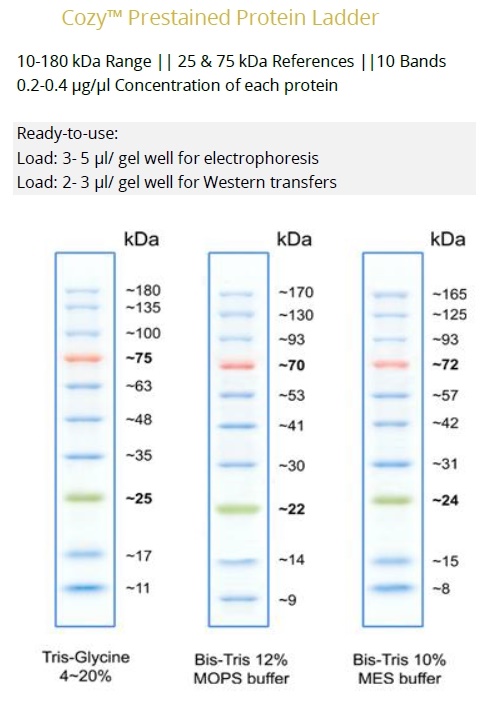


Figure 24: Cozy™ Prestained Protein Ladder

Link: https://www.highqu.com/Cozy-Prestained-Protein-Ladder/PRL0102

## Dual Luciferase Reporter Assay Solutions - Hampf and Gossen (2)

**Prepare 2X solutions A and B:**

| **2X solution A (Firefly)** | | |  | **2X solution B (Renilla)** | | |
| --- | --- | --- | --- | --- | --- | --- |
| 2 M | **Tris-HCL** | 20 ml |  |  | **Na_2_SO_4_** | 14.2g |
| 150 mM | **MgSO_4_** | 20 ml |  | 5 M | **NaCl** | 20 ml |
| 10 mM | **EDTA** | 2 ml |  | 150 mM | **EDTA** | 20 ml |
|  |  |  |  | 100 mM | **NaAc** | 20 ml |
|  | **H_2_O** | 38 ml |  |  | **H_2_O** | 30 ml |
| 🡒 adjust to **pH 8.0** | | |  | 🡒 adjust to **pH 5.0** | | |
| 🡒 fill up to 100 ml | | |  | 🡒 fill up to 100 ml | | |

**Keep both 2X solutions at 4°C.**

**Prepare 1X solutions A and B for measurement**

| **1X solution A (Firefly)** | | 20 ml | **10 ml** | 5 ml |
| --- | --- | --- | --- | --- |
| 2X | **solution A** | 10 ml | **5 ml** | 2.5 ml |
| 250 mM | **DTT** | 2 ml | **1 ml** | 500 µl |
| 10 mM | **ATP** | 2 ml | **1 ml** | 500 µl |
|  | **H_2_O** | 4 ml | **2 ml** | 1 ml |
| *🡒 keep at -20°C* | | | | |
| *🡒 freshly add before measurement:* | | | | |
| 2 mM | **Luciferin** | 2 ml | **1 ml** | 500 µl |
|  |  |  |  |  |
| **1X solution B (Renilla)** | | 20 ml | **10 ml** | 5 ml |
| 2X | **solution B** | 10 ml | **5 ml** | 2.5 ml |
| 250 mM | **Na_2_PP_i_** | 2 ml | **1 ml** | 500 µl |
|  | **H_2_O** | 5.8 ml | **2.9 ml** | 1.45 ml |
| *🡒 keep at -20°C* | | | | |
| *🡒 freshly add before measurement:* | | | | |
| 500 µM | **APMBT (in H_2_O)** | 2 ml | **1 ml** | 500 µl |
| 400 µM | **Coelenterazine (in Methanol)** | 200 µl | **100 µl** | 50 µl |

**Prewarm the solutions to room temperature before starting the measurement and keep them in the dark whenever possible.**

**Supplementary Material 2: SOP for HeLa cultivation in CDM**

Standard Operating Procedure (SOP) for the

**Adaptation and Cultivation of HeLa Cells in Chemically Defined Medium**

Content

[I. HeLa_Preparation of media and reagents 2](#_Toc198122870)

[II. HeLa_direct adaptation to CDM medium 9](#_Toc198122871)

[III. HeLa_culturing in CDM medium 9](#_Toc198122872)

[IV. HeLa_freezing in chemically defined freezing medium 12](#_Toc198122873)

[V. HeLa_thawing cells frozen in chemically defined freezing medium 14](#_Toc198122874)

### HeLa_Preparation of media and reagents

**1. Introduction**

This protocol outlines the preparation of media and reagents necessary for the cultivation, passaging, and cryopreservation of HeLa cells under chemically defined, serum-free conditions. For general information about animal cell culture techniques, the reader is referred to the excellent introduction by Mani et al. (1).

| **Media and reagents:**  DMEM/ F12 (**Biowest, L0090**)  Non-Essential Amino Acids 100X (NEAA) (**Biowest, X0557 - 100**)  4-(2-Hydroxyethyl)-piperazin-1-ethansulfonsäure (HEPES) (**Biowest, L0180**)  Glutamin (100x) (**Biowest, X0550**)  D-(+)-glucose (**Sigma, G8769**)  Insulin-Transferrin-Selenium (100x) (ITS) (**Gibco, 41400045**)  Recombinant human epidermal growth factor (hEGF) (**Gibco, PHG0313**)  Hydrocortisone 21-hemisuccinate sodium salt (HC) (**St Cruz Biotech, sc-250130**)  Dulbecco’s phosphate buffered saline (DPBS) (**Biowest, L0615**)  TrypLE^TM^ Express (**Gibco, 12604021**) or TrypLE^TM^ Select (**Gibco, 12563029**)  Trypsin inhibitor from glycine max (soybean) (**Sigma, 93620-250MG**)  Dimethyl sulfoxide (**DMSO, Sigma, D2650**)  Distilled water  **Materials and equipment:**  Cell culture flasks (**TPP**)  5ml/10ml/25ml pipettes  Aspiration pump  1.5ml Eppendorf tubes (sterile)  Falcon tubes (15 ml/50ml)  Balance |
| --- |

**2. Storage of media and reagents**

Ensure that media/reagents are stored properly upon delivery from suppliers (see table below).

| **Media/reagent** | **Storage temperature (°C)** | | |
| --- | --- | --- | --- |
|  | **RT** | **4°C** | **-20°C** |
| DMEM/ F12 |  | ● |  |
| NEAA |  | ● |  |
| HEPES |  | ● |  |
| Glutamin (100x) |  | ● |  |
| D-(+)-glucose | ● |  |  |
| ITS |  | ● |  |
| hEGF |  |  | ● |
| Hydrocortisone |  |  | ● |
| DPBS | ● |  |  |
| TrypLE^TM^ Express | ● |  |  |
| Trypsin inhibitor |  |  | ● |
| DMSO | ● |  |  |

**3. Making aliquots**

**hEGF**: Prepare aliquots of 50-100 μl (100 μg/ml in sterile water) and store at -20 °C.

**HC**: Prepare aliquots of 20 μl (10 μg/ml in sterile water) and store at -20 °C

**TrypLE^TM^ Express**: Prepare 50 mL aliquots to avoid contamination of stock solution. Store at RT.

**Trypsin inhibitor:** Prepare Stock of 10 mg/ml (1%) in DPBS and sterile-filtrate. Aliquot into 1 ml and store at -20 °C

**4. Preparation of CDM base medium**

| **Component** | **Stock**  **concentration** | **Final**  **concentration** | **≈ 500 mL** | **Notes** |
| --- | --- | --- | --- | --- |
| DMEM/ F12 | Appendix 1 | - | 500 ml |  |
| NEAA | 100x | 1x | 5 ml |  |
| HEPES | 1 M | 15 mM | 7.5 ml |  |
| D-(+)-glucose | 45% | 0.1% | 1.1 ml |  |

Store the prepared CDM base medium at 4 °C.

**5.** **Preparation** **of 10x supplement solution**

| **Component** | **Stock**  **concentration** | **Final**  **concentration** | **50 mL** | **Notes** |
| --- | --- | --- | --- | --- |
| Glutamin | 100x | 10x | 5 ml |  |
| ITS | 100x | 10x | 5 ml |  |
| hEGF | 100 μg/ml | 100 ng/ml | 0.05 ml |  |
| HC | 50 mg/ml | 10 µg/ml | 0.01 ml |  |

Store the solution in aliquots at -20°C.

**6. Preparation of complete CDM medium**

To prepare complete medium, add 1/10 volume of supplement solution freshly to the required volume of CDM base medium. The prepared medium can be stored at 4 °C for at least one month.

**7. Preparation of TrypLE inactivating solution (TIS)**

7.1 Allow the aliquoted trypsin inhibitor to equilibrate at room temperature for 3-5 minutes.

7.2 Prepare the TIS as indicated in table below.

For example:

| **Component** | **Stock**  **Concentration** | **Final**  **concentration** | **20 mL** |
| --- | --- | --- | --- |
| CDM base medium | See 4. | - | 19 ml |
| Trypsin inhibitor | 10 mg/mL (~ 10000 U/mg) | 0.5 mg/mL | 1 ml |

7.3 Store the prepared solution at 4 °C for 1-2 weeks.

**8. Preparation of chemically defined freezing medium (CDFM)**

8.1 Prepare CDFM as indicated in table below.

| **Component** | **Stock**  **Concentration** | **Final**  **concentration** | **50 mL** |
| --- | --- | --- | --- |
| Culture medium (CDM) | (Current protocol) | - | 45 ml |
| DMSO | 100% | 10% | 5 ml |

8.2 Prepare freezing medium freshly before use.

8.3 Pre-warm the aliquoted medium to 37 °C immediately before cell cryopreservation.

**Appendix 1**

(Taken from: https://biowest.net/wp-content/uploads/2022/12/l0090-tds.pdf)


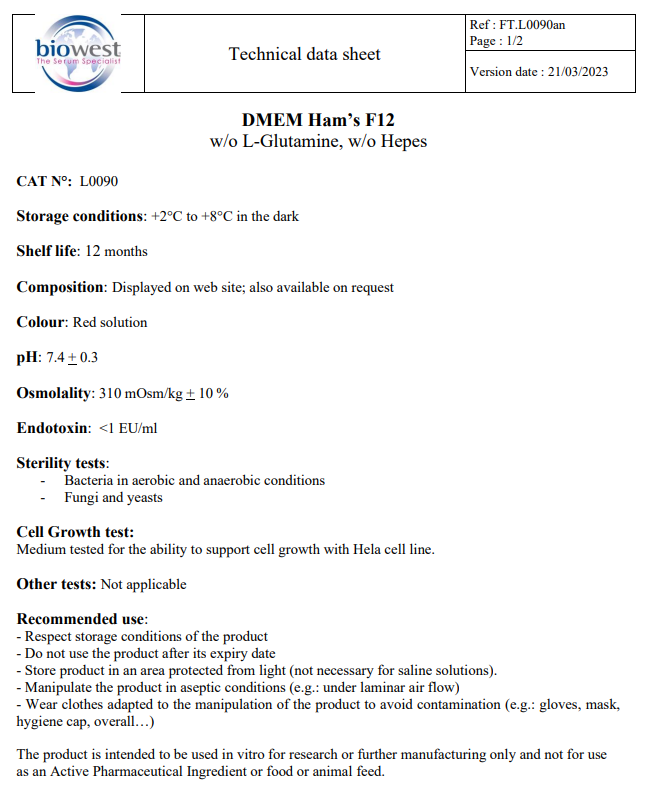


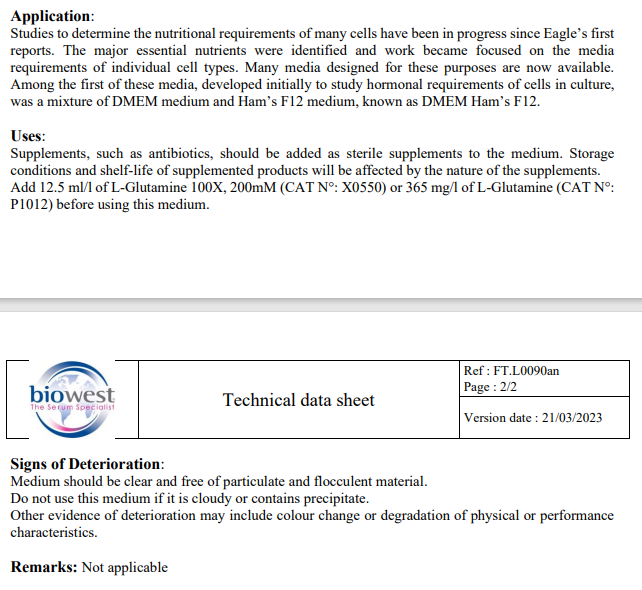


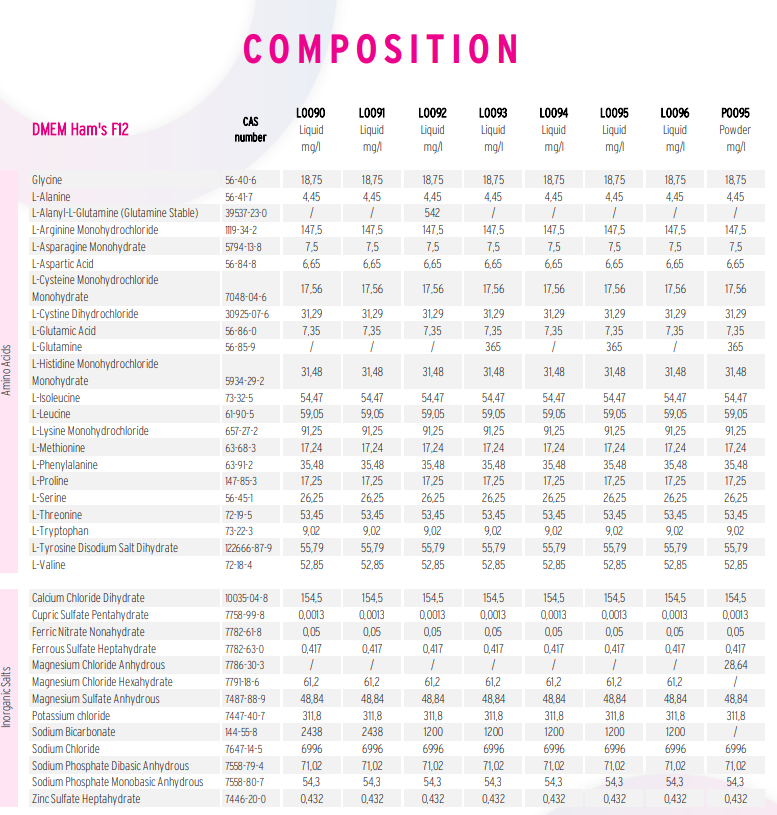


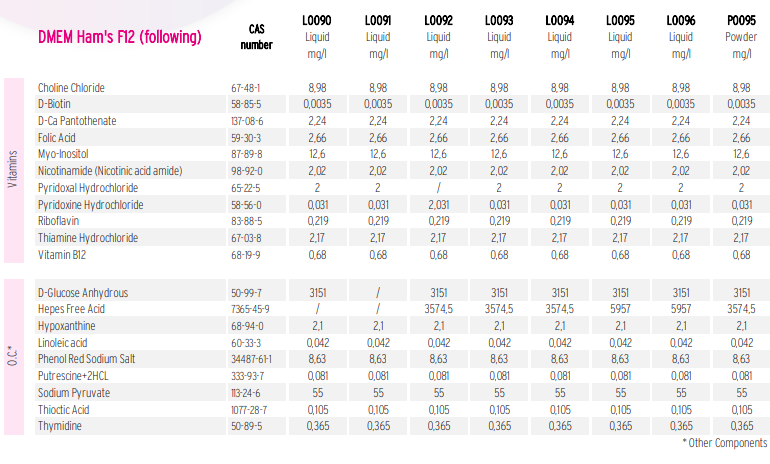


### HeLa_direct adaptation to CDM medium

**Media and reagents:**

Chemically defined medium (CDM) (**See section I**)

TrypLE^TM^ Express (**Gibco, 12604021**) or TrypLE^TM^ Select (**Gibco, 12563029**)

TrypLE inactivating solution (TIS) (**See section I**)

Dulbecco’s phosphate buffered saline (DPBS) (**Biowest, L0615**)

**Materials and equipment:**

Cell culture flasks **(TPP)**

5 ml / 10 ml / 25 ml pipettes

Aspiration pump

Conical tubes (15 ml/ 50 ml)

**1. Introduction**

This protocol outlines the direct transition of HeLa cells from culture in medium supplemented with 10% fetal bovine serum (FBS) to chemically defined medium (CDM) that is entirely free of serum and other animal-derived components, enabling fully animal-free culture conditions.

The method employs a direct adaptation approach, eliminating the need for gradual reduction of FBS concentrations over successive passages. Upon switching to CDM, no significant morphological changes were observed between the initial and subsequent passages.

To minimize the influence of residual intracellular FBS, it is recommended to subculture HeLa cells for at least six consecutive passages in CDM before cryopreservation or use in downstream applications.

**2.** **Procedure**

2.1 Thaw HeLa cells and transfer them into a T25 flask containing FBS-supplemented medium. Incubate at 37 °C with 5% CO₂ for initial recovery (refer to thawing procedure in Section V).

2.2 Cultivate cells until they reach high confluency (80–100%). Ensure cells exhibit a high proliferative rate before initiating the switch to CDM.

2.3 For the first passage in CDM, split the cells at a 1:2 ratio into a new T25 flask using CDM (refer to Section I, Steps 1–6).

2.4 Incubate at 37 °C with 5% CO₂.

2.5 Replace the medium every 2–3 days.

2.6 Once cells reach 80–100% confluency, passage them again using a 1:2 or 1:3 split ratio (see procedure in Section III).

2.7 Continue subculturing in CDM for at least six consecutive passages.

2.8 After six passages in CDM, cells can be considered fully adapted to FBS-free conditions.

### HeLa_culturing in CDM medium

| **Media and reagents:**  Chemically defined medium (CDM) (**See section I**)  TrypLE^TM^ Express (**Gibco, 12604021**) or TrypLE^TM^ Select (**Gibco, 12563029**)  TrypLE inactivating solution (TIS) (**See section I**)  Dulbecco’s phosphate buffered saline (DPBS) (**Biowest, L0615**)  **Materials and equipment:**  Cell culture flasks **(TPP)**  5 ml / 10 ml / 25 ml pipettes  Aspiration pump  Conical tubes (15 ml/ 50 ml) |
| --- |

**1. Cell culture procedure**

1.1 Set up the water bath and preheat to 37 °C.

1.2 Pre-warm the required volume of CDM (see Protocol 1.04). Refer to the table below for vessel-specific volumes.

| Culture vessel | Surface area (cm^2^) | Volume of medium |
| --- | --- | --- |
| T-150 flask | 150 | 25 ml per flask |
| T-75 flask | 75 | 20 ml per flask |
| T-25 flask | 25 | 5 ml per flask |

1.3 Pre-warm DPBS, TrypLE, and TrypLE Inactivation Solution (TIS) (see Protocol 1.04).

1.4 Aspirate the spent medium from the culture flask using a sterile pipette connected to an aspiration system.

1.5 Rinse cells once with the appropriate volume of pre-warmed DPBS (see table below).
1.6 Add the recommended volume of TrypLE (see table) and incubate for 3–5 minutes at 37 °C.
1.7 Inactivate TrypLE by adding the appropriate volume of TIS. Gently pipette up and down to detach and collect the cells.

| Culture vessel | Surface area (cm^2^) | Volume of DPBS | Volume of TrypLE | Volume of TIS |
| --- | --- | --- | --- | --- |
| T-150 flask | 150 | 10 mL per flask | 3 mL per flask | 6 ml |
| T-75 flask | 75 | 7 mL per flask | 1.5 mL per flask | 3 ml |
| T-25 flask | 25 | 5 mL per flask | 0.5 mL per flask | 1 ml |

1.8 Transfer the cell suspension to a 15 mL or 50 mL centrifuge tube and centrifuge at 300 × g for 3 minutes at room temperature.
1.9 Carefully discard the supernatant without disturbing the cell pellet.
1.10 Resuspend the cell pellet in CDM to obtain a concentrated cell suspension.
1.11 Transfer the appropriate volume of cell suspension (based on the desired split ratio) into new culture vessels containing fresh CDM.
  **Note:** A typical split ratio is 1:2 to 1:4. Subculturing is usually required 2–3 times per week.
1.12 Gently rock the culture vessel side-to-side and front-to-back to ensure even distribution of cells.
1.13 Document all relevant details, including the date, cell line, passage number, and split ratio.
1.14 Incubate the cells in a humidified incubator at 37 °C with 5% CO₂ until the next passage or experiment.
1.15 If cells are not ready for splitting after 72 hours or the experiment is prolonged, replace the medium.
1.16 Subculture cells during the logarithmic growth phase (typically at 80–90% confluency). Avoid waiting until 100% confluency.
1.17 For seeding in multiwell plates, refer to the volume table below.

| Culture vessel | Surface area (cm^2^) | Volume of medium |
| --- | --- | --- |
| 6-well plate | 10 | 2 ml per well |
| 12-well plate | 3.5 | 1 ml per well |
| 24-well plate | 1.9 | 500 µl per well |
| 48-well plate | 1.1 | 250 - 300 µl per well |
| 96-well plate | 0.32 | 100 - 200 µl per well |

### HeLa_freezing in chemically defined freezing medium

| **Media and reagents:**  Chemically defined freezing medium (CDFM) (**See section I**)  TrypLE^TM^ Express (**Gibco, 12604021**)  TrypLE inactivating solution (TIS) (**See section I**)  Dulbecco’s phosphate buffered saline (DPBS) (**Biowest, L0615**)  **Materials and equipment:**  5 ml / 10 ml / 25 ml pipettes  Aspiration pump  Falcon tubes (15 ml / 50 ml)  Freezing container (Mr. Frosty) filled with 100% 2-propanol  Pre-labeled 2 ml cryo vials |
| --- |

**1. Introduction**

This protocol outlines the cryopreservation of HeLa cells using a chemically defined freezing medium (CDFM) for long-term storage in liquid nitrogen under serum-free, animal-free conditions.

**2. Cryopreservation procedure**

2.1 Cryopreserve only cells in the logarithmic growth phase, typically at 80–90% confluency.

2.2 Pre-warm TrypLE inactivating solution (TIS), freezing medium (CDFM), DPBS and TrypLE Express at 37 °C.

2.3 Prepare cryo vials by labelling them with the date, cell line, passage number, and freezing media information.

2.4 Aspirate medium from the culture vessel and rinse once with appropriate volume of pre-warmed DPBS (see table below).

2.5 Add appropriate volume of TrypLE to the culture vessels (see table below) and incubate for 3 – 5 minutes at 37 °C.

2.6 Inactivate TrypLE by adding the appropriate volume of pre-warmed TIS (see table). Gently pipette the suspension up and down to collect all detached cells

| Culture vessel | Surface area (cm^2^) | Volume of DPBS | Volume of TrypLE | Volume of TIS |
| --- | --- | --- | --- | --- |
| T-150 flask | 150 | 10 ml per flask | 3 ml per flask | 6 ml |
| T-75 flask | 75 | 7 ml per flask | 1.5 ml per flask | 3 ml |
| T-25 flask | 25 | 5 ml per flask | 0.5 ml per flask | 1 ml |

2.7 Count cells (using a Neubauer counting chamber) and calculate cell concentration.

2.8 Transfer the required volume of cell suspension into a sterile centrifuge tube.

2.9 Centrifuge at 300 x g for 3 minutes at room temperature.

2.10 Discard the supernatant carefully without destroying the cell pellet.

2.11 Resuspend the cell pellet in appropriate volume of CDFM to obtain a concentrated cell suspension (1.5x10^6^ cells/ml).

2.12 Add 1 mL of cell suspension (1.5x10^6^ cells) to each cryovial, ensuring consistent cell concentration between vials.

2.13 Cap the cryo vials tightly and ensure they are leak-proof.

2.14 Place the cryo vials immediately into the freezing container and place at -80°C overnight.

2.15 Transfer the cells to a liquid nitrogen tank the next day.

### HeLa_thawing cells frozen in chemically defined freezing medium

| **Media and reagents:**  Chemically defined medium (CDM) (**See protocol 1.04**)  **Materials and equipment:**  T-25 culture flask (**TPP**)  5 mL pipettes |
| --- |

**1. Introduction**

This protocol outlines the proper thawing procedure for HeLa cells cryopreserved in chemically defined freezing medium (CDFM) to ensure maximum viability and recovery under serum- and animal-free conditions.

**2. Thawing procedure**

2.1 Set up a sterile 37 °C water bath and pre-warm 5 mL of chemically defined medium (CDM).
2.2 Pipette 4 mL of the pre-warmed CDM into a sterile T25 culture flask.
2.3 Carefully remove the desired cryovial from the liquid nitrogen tank, avoiding unnecessary exposure of other vials to temperature fluctuations.
2.4 Transfer the vial to the laminar air flow bench, briefly open the cap to release any built-up pressure, then immediately place the vial into the 37 °C water bath.
2.5 Gently swirl the vial in the water bath until fully thawed (typically 1–2 minutes). Avoid vigorous shaking to minimize cell damage.
2.6 Remove the vial from the water bath and disinfect the exterior with 70% ethanol.
2.7 Using a sterile pipette, add 1 ml of pre-warmed CDM to the thawed cell suspension.
2.8 Transfer the entire contents of the vial into the prepared T25 flask by slowly pipetting the suspension directly into the medium.
2.9 Gently rock the flask side to side and front to back to evenly distribute the cells across the surface.
2.10 Incubate the flask at 37 °C in a humidified atmosphere with 5% CO₂.
2.11 After 24 hours, replace the medium with fresh CDM to remove residual DMSO and promote healthy cell recovery.

*Reference*

1. Mani S, Singh M, Kumar A. Animal Cell Culture: Principles and Practice. Kalyuzhny AE, editor. Cham, Switzerland: Springer Nature Switzerland 2023.

**Supplementary Material 3: Transcript of Surveys**

Lab Courses 2025

(Answers in German were translated into English, and some edits were made to improve clarity. Otherwise, the student comments were left as they were.)

1. What grade would you give the course?

Average grade: 1.5 (on a scale of 1 (best) to 6 (worst)), n=21

1. What do you think is a good group size for the practical course? (Please be reminded that we want to be able to enroll as many students as we have room for.)

Grade: 2.4

- Two students at the laminar flow bench, 1 student gives instructions (Procedure according to the script)
- It was a good group size.
- 10-15; no more than this; 10 was perfect.

1. Did you find the number and combination of experiments straightforward and appropriate? Grade: 1.5

What can be improved?

- Good combination of experiments, but the coordination with the other groups could be better.
- It is definitively good and better than finishing each experiment and having to wait for long time.
- Detailed timeline for the groups (make sure that none of the groups is done earlier than the others)
- Upload the script earlier
- Yes, but the time schedule could be improved.
- More separation of the experiments (even though it’s hard to do)
- Combination was great; definitively not more experiments.
- Combination is really good, but the time schedule could be improved; sometimes long breaks for some groups.
- Actually, I thought that a fixed, arranged, or more coordinated sequence would be better, but everything has always worked out very well.
- Less spare time if tutors are free, but experimental pipeline was good.

1. Did you feel well looked after during the experiments?
   Grade: 1.2 (on a scale of 1 to 5)

What can be improved?

- Clearer information if you should wait to be picked up.
- Especially on the first day we had a lot of time before the first experiment for the calculations & the tutors were all busy with other groups so there was no one to ask questions in that moment which cost time. During the experiments in the lab, it was great.
- All tutors and supervisors were very motivated, and it was a pleasure working with them.
- I felt over-monitored during the experiments.

1. How did you find the script/course material?
   Grade: 1.9 (on a scale of 1 to 5)
   What can be improved?

- Tiny errors in script that led to some confusion, overall good explanations.
- The script was written really good, and everything was explained precisely.
- Complete the script, include duplicates/triplicates of the experiments
- More details on each step & purpose of Western blot experiment; upload the script earlier
- Tables could be improved; sometimes they were chaotic.
- The tables for the calculations.
- Just some small corrections of the script (tables); add flowchart for the DLA.
- More accurate materials / what was used.
- The tables
- Tables!!! Also, the texts were a bit confusing sometimes.
- Tables and some flow charts were irritating.
- The tables were confusing.
- The tables with the concentrations need to be improved. They are so confusing.
- It would be helpful if the script could be uploaded earlier. Sometimes it was a bit confusing, especially the tables.
- Tables were confusing.
- The K4 experiment calculations in the table.
- The time schedule
- Tables; the experimental schemes.
- The tables regarding the calculation of concentrations are not intuitive.

1. What did you like about the course?

- Learning material, supervision
- That it was (almost) animal free! The supervisors did a great job.
- I learned something new, new methods; I liked the presentation day, so we had the chance to see what others have done during the week.
- Script, friendly supervision, competent
- Structure, Explanation
- Lots of new methods, small groups, very hands-on and updated methods; everything was thematically related; already some lab on Monday (usually its only intro + safety)
- The supervisors
- Very lovely people and a good structure, was a lot of fun.
- Interesting experiments with new handling mechanics (for me personally); lots of methods.
- I learned a lot + never felt like I was being judged for not doing everything perfectly; group protocol!!! So nice. Loved that we had many different tutors, it was cool to see different teaching styles.
- Interesting experiments; good balance of workload; feeling looked after.
- I learned a lot due to the number of different experiments.
- The supervisors were really kind and knowledgeable. Took the time to explain everything in detail. Was fun learned a lot of new techniques (in depth).
- The script was informative and straight to follow; Lectures were understandable; easy to follow; ppt-slides good to study; the experiments were well-planned and supervised; supervisors were always friendly and helpful.
- I liked that the experiments all basically belonged together. The group size was perfect; lot of time in the lab.
- Nice people, self-directed timing, and RNA interference as the topic.
- A lot and very different experiments.
- I loved it! Thank you so much for a well-organized course and a very nice script. Thank you for always answering questions.
- The tutors.
- Very interesting experiments, I learned a lot; small group size helped a lot to gain much practical experience; the we had time to prepare the presentation.
- Amazing tutors; interesting experiments and thorough discussion of results.

1. What did you not like about the course?

- Waiting time on day 1 (use the time for things like filling tip boxes); Script was uploaded too late. Sometime long breaks; on other days it was well structured.
- Organization, the timetable in the script was not always followed.
- The waiting time after each experiment was too long and it was a little bit unorganized.
- Lots of waiting times with nothing to do; no results yet; intro to presentation is done in 30 min, no information on the protocol
- Time schedule was chaotic (only sometimes)
- For group 1 the qPCR results should have been discussed earlier.
- Some unfortunate downtimes due to the on clean bench.
- That we had to be there at 9 a.m.
- The tables in the script.
- Organization and script. I would have wished that the schedule for the experiments would have been fixed and not changed on the first day.
- The time to prepare the presentation was a little short (if you didn’t have time to work at home).
- Please upload the lectures earlier OR do a lecture per week throughout the semester.
- Sometimes the time management; sometimes I felt a little “grilled” on questions which I couldn’t answer even though I read the script (but in general I think it was good that we talked a lot about the experiments)
- Sometimes long breaks; no room for us; sitting in the corridor is not so nice.
- Based on the announcements at the beginning, I thought we would have much more time in-between to prepare presentations and study for the exam. Instead, we were almost constantly involved in different experiments, which was actually a good thing. However, the preparation for the presentation felt quite hectic as a result.
- It only takes place in Winter semester.
- Days were very long; transitions between experiments were sometimes too quick and confusing; that we have to write a protocol for all experiments.
- Sitting in the corridor; to be monitored too closely, have more confidence in us.
- Limited time for preparation of presentations.

1. What do you think we should consider for future courses, what would you like to see?

- Don’t know; liked it
- Plasmid prepping
- Maybe more individual work; sometimes performing an experiment while 2-4 persons observe can be stressful and lead to experiment errors.
- Maybe extend the time of the course to two weeks.
- BCA assay for Western blot, maybe comparison of qPCR (SYBR green) and Taqman (if not to expensive); Comparison of SYBR green and ethidium bromide or something like that.
- Group size to two people; I know it is not really an option.
- Hard to tell; it was my favorite lab course so far and improved on a lot that I usually don’t like.
- Explain in more detail the reasoning for experiments in big picture context.
- I thought maybe some sequencing would be cool, but I know that it is hard to organize.
- Do it the same way!
- Explain more about the context for the experiments, maybe especially in the beginning because the script itself is a lot of input & it helps if someone says what the scope of the course is & how the experiments belong together.
- Maybe upload the script earlier.
- Earlier upload of material.
- More breaks for the tutors.
- Viral vectors.
- Maybe 7-day practical course with shorter lab days.

1. Further information and suggestions:

- Earlier information on protocol to have something to work on. Or more example exercises. Grade weights do not match with ECTS. Maybe at least 50% of the grade should be from practical course (6 ECTC) and 50% from examine (3 ECTS)
- Stay the way you are.
- Better script for the next courses.
- Remove preliminary questionnaire
- It was very nice and interesting, thank you.

**Supplementary Material 4: Cost of the Culture Media**

Price estimate for conventional HeLa medium supplemented with 10% FBS compared to Chemically Defined Medium. Prices are based on the conditions for the Technische Universität Berlin as of May 2025.

| **Conventional HeLa**  **Medium** |  |  |  |  |  |  |  |  |
| --- | --- | --- | --- | --- | --- | --- | --- | --- |
| **Component** | **Article** | **Composition** | **f.c.** | **Price €**  **netto** |  | **Distributor** | **Unit of Sale** | **Price €**  **netto** |
| DMEM (low glucose) | BioWest L0064 | 500 ml |  | 9.80 |  | VWR | 500 ml | 9.80 |
| FBS | c-c-pro | 50 ml | 10% | 19.25 |  | c-c-pro | 500 ml | 192.50 |
| L-Glutamin | BioWest X0550 | 5,5 ml | 2 mM | 0.64 |  | VWR | 100 ml | 11.70 |
| NEAA | BioWest X0557 | 5.5 ml | 1x | 0.96 |  | VWR | 100 ml | 17.50 |
| total |  |  | Total netto | 30.65 |  |  |  |  |
|  |  |  |  |  |  |  |  |  |
| **Hela CDM** |  |  |  |  |  |  |  |  |
| **Component** | **Article** | **Composition** | **f.c.** | **Price €**  **netto** |  | **Distributor** | **Unit of Sale** | **Price €**  **netto** |
| DMEM/ F12 | BioWest L0090 | 500 ml |  | 12.20 |  | VWR | 500 ml | 12.20 |
| NEAA | BioWest X0557 | 5 ml | 1x | 0.88 |  | VWR | 100 ml | 17.50 |
| HEPES | BioWest L0180 | 7.5 ml | 15 mM | 2.00 |  | ThGeyer | 100 ml | 26.60 |
| Glutamin | BioWest X0550 | 5 ml | 2 mM | 0.57 |  | VWR | 100 ml | 11.70 |
| D-Glucose | Sigma G8769 | 1.1 ml | 0,10% | 0.59 |  | SigmaAldrich | 100 ml | 44.32 |
| ITS | Gibco 41400045 | 5 ml | 1x | 33.25 |  | Thermo Fisher Scientific | 10 ml | 66.50 |
| hEGF | Gibco PHG0313 | 2.5E-3 ml | 10 ng/ml | 3.54 |  | Thermo Fisher Scientific | 1 mg | 676.60 |
| Hydrocortisone 21-hemisuccinate sodium salt | St Cruz Biotech, sc-250130 | 0.5E-3 ml | 1 µg/ml | 0.25 |  | St Cruz Biotechnology | 100 mg | 48.00 |
| total |  |  | Total netto | 53.28 |  |  |  |  |
